# Multiple Myeloma Cells with Increased Proteasomal and ER Stress Are Hypersensitive to ATX-101, an Experimental Peptide Drug Targeting PCNA

**DOI:** 10.3390/cancers16233963

**Published:** 2024-11-26

**Authors:** Camilla Olaisen, Lisa Marie Røst, Animesh Sharma, Caroline Krogh Søgaard, Tiffany Khong, Sigrid Berg, Mi Jang, Aina Nedal, Andrew Spencer, Per Bruheim, Marit Otterlei

**Affiliations:** 1 Department of Clinical and Molecular Medicine, Faculty of Medicine and Health Sciences, NTNU Norwegian University of Science and Technology, NO-7491 Trondheim, Norway; camilla.olaisen@stolav.no (C.O.); caroline.d.sogaard@ntnu.no (C.K.S.); sigrbe@ntnu.no (S.B.); aina.nedal@ntnu.no (A.N.); 2 Department of Biotechnology and Food Science, Faculty of Natural Sciences, NTNU Norwegian University of Science and Technology, NO-7491 Trondheim, Norway; lisa.marie.rost@gmail.com (L.M.R.); kia3111@gmail.com (M.J.); per.bruheim@ntnu.no (P.B.); 3 Proteomics and Modomics Experimental Core Facility (PROMEC), NTNU Norwegian University of Science and Technology, NO-7491 Trondheim, Norway; animesh.sharma@ntnu.no; 4 Australian Centre for Blood Diseases, Monash University, Melbourne 3004, Australia; tiffany.khong@monash.edu (T.K.); andrew.spencer@monash.edu (A.S.); 5 Department of Malignant Haematology and Stem Cell Transplantation, Alfred Hospital, Melbourne 3004, Australia; 6 Clinic of Surgery, St. Olavs Hospital, Trondheim University Hospital, NO-7006 Trondheim, Norway; 7 APIM Therapeutics A/S, Rådhusveien 12, NO-7100 Rissa, Norway

**Keywords:** redox status, ribosomal gene expression, glycolysis, PPP, metabolites

## Abstract

**Objectives**: To examine the regulatory role of PCNA in MM, we have targeted PCNA with the experimental drug ATX-101 in three commercial cell lines (JJN3, RPMI 1660, AMO) and seven in-house patient-derived cell lines with a more primary cell-like phenotype (TK9, 10, 12, 13, 14, 16 and 18) and measured the systemic molecular effects. **Methods**: We have used a multi-omics untargeted approach, measuring the gene expression (transcriptomics), a subproteomics approach measuring mainly signalling proteins and proteins in complex with these (signallomics) and quantitative metabolomics. These results are supplemented with traditional analysis, e.g., viability, Western and ELISA analysis. **Results**: The sensitivity of the cell lines to ATX-101 varied, including between three cell lines derived from the same patient at different times of disease. A trend towards increased sensitivity to ATX-101 during disease progression was detected. Although with different sensitivities, ATX-101 treatment resulted in numerous changes in signalling and metabolite pools in all cell lines. Transcriptomics and signallomics analysis of the TK cell lines revealed that elevated endogenous expression of ribosomal genes, elevated proteasomal and endoplasmic reticulum (ER) stress and low endogenous levels of NAD+ and NADH were associated with ATX-101 hypersensitivity. ATX-101 treatment further enhanced the ER stress, reduced primary metabolism and reduced the levels of the redox pair GSH/GSSG in sensitive cells. Signallome analysis suggested that eleven proteins (TPD52, TNFRS17/BCMA, LILRB4/ILT3, TSG101, ZNRF2, UPF3B, FADS2, C11orf38/SMAP, CGREF1, GAA, COG4) were activated only in the sensitive MM cell lines (TK13, 14 and 16 and JJN3), and not in nine other cancer cell lines or in primary monocytes. These proteins may therefore be biomarkers of cells with activated proteasomal and ER stress even though the gene expression levels of these proteins were not elevated. Interestingly, carfilzomib-resistant cells were at least as sensitive to ATX-101 as the wild-type cells, suggesting both low cross-resistance between ATX-101 and proteasome inhibitors and elevated proteasomal stress in carfilzomib-resistant cells. **Conclusions**: Our multi-omics approach revealed a vital role of PCNA in regulation of proteasomal and ER stress in MM.

## Simple Summary

In addition to being essential for DNA replication and repair, proliferating cell nuclear antigen (PCNA) has recently been linked to regulation of signalling, metabolism and apoptosis. As PCNA is a new and unexplored target for cancer treatment, we have here investigated the regulatory role of PCNA in 10 multiple myeloma (MM) cell lines using a multi-omics approach and a PCNA-targeting peptide, ATX-101, currently in clinical development. We have found that ATX-101’s efficacy is linked to PCNA’s role in regulation of proteasomal and ER stress in MM cells.

## 1. Introduction

PCNA is essential for DNA replication, repair and damage tolerance [1], but recent data have shown that PCNA also acts as a scaffold in metabolism and is important for regulation of apoptosis, immune responses and cellular signalling [2,3,4,5,6]. More than 600 mammalian proteins may potentially interact with PCNA via the two known PCNA-interacting motifs, AlkB homologue 2 PCNA-interacting motif (APIM) or the PCNA-interacting peptide box (PIP-box) [7,8,9]. Therefore, binding to PCNA must be regulated by multi-layered mechanisms including different post-translational modifications (PTMs) on PCNA and large variations in binding affinities within and between the two interaction motifs [1,10]. The canonical PIP-boxes have the highest affinity for PCNA under normal conditions [3,11], while the affinity for APIM and non-canonical PIP-boxes increases with PTMs on PCNA [8,12]. Proteins essential for DNA replication have high affinity for canonical PIP-box motifs, while proteins important for the handling of cellular stress often contain APIM or non-canonical PIP-boxes. For example, a cell-penetrating peptide containing APIM (ATX-101) interacts with PCNA and blocks protein–PCNA interactions after cellular stress but does not inhibit normal replication [5,13]. This suggests that mainly stress-mediated scaffold roles of PCNA are targeted by the peptide. In support of this, ATX-101 is shown to increase the efficacy of DNA-, microtubule- and kinase-targeting anticancer drugs in pre-clinical murine cancer models [5,13,14,15], to reduce DNA repair and damage tolerance [8,16,17] and to alter the regulation of cellular signalling, metabolism and apoptosis [3,6,13,14]. In a recent Phase I study, ATX-101 was shown to be well tolerated with no grade 3 or 4 adverse effects, and the absence of, for example, myelosuppression supports that ATX-101 mainly targeted stressed and not normal replicating cells. Interestingly, a large fraction (~70%) of the efficient patient population with advanced cancer had stable disease at the end of the study, indicating the biological activity of targeting the PCNA-regulated stress responses [18].

Seven glycolytic enzymes catalysing steps 4–10 in the glycolytic pathway were early on reported to be in complex with PCNA, i.e., aldolase, triosephosphate isomerase, glyceraldrehyd-3-phosphate dehydrogenase (GAPDH), phosphoglycerate kinase, phosphoglycerate mutase (PGAM1), enolase (ENO1) and pyruvate kinase (PKM) [19]. However, whether these interactions were direct or indirect, or the function of these interactions, was not known. Recently, it was shown that mutation of one amino acid in the PCNA interaction motif APIM in ENO1 affected ENO1’s ability to bind PCNA and its protein stability [6]. This led to reduced glucose consumption and a metabolic shift characterised by accumulation in glycolytic metabolite pools above ENO1 and reduced metabolite pools below ENO1. Further, ENO1 mutated cells showed reduced growth and reduced AKT signalling activity compared to parental cells. ENO1 is overexpressed in multiple cancers, including MM, and is regarded as a potential target for cancer therapy [20,21].

In addition to the glycolytic enzymes, another metabolic enzyme, 6-phosphogluconate dehydrogenase (6PGD), which catalyses the third step in the pentose phosphate pathway (PPP), also contains a putative PCNA-interacting motif [8]. 6PGD is frequently overexpressed in cancers, and the inhibition of 6PGD-activity is shown to impair cancer growth [22,23]. The protein levels of both 6PGD and ENO1, the glucose consumption levels of glycolytic and PPP intermediates and nucleoside triphosphate (NTP) pools were reduced in multiple haematological cell lines including two MM cell lines after ATX-101 treatment [6]. This metabolic shift was not detected in untreated primary monocytes from healthy donors; however, if the monocytes were concurrently stimulated with the toll-like receptor (TLR) ligand 4 (lipopolysaccharide, LPS), the levels of glycolytic and PPP intermediates and the nucleoside triphosphate (NTP) pools were reduced similarly to the haematological cancer cell lines [6]. This supports that cellular stress levels are important for the efficacy of ATX-101.

MM is an incurable cancer of plasma cancer cells that accounts for approximately 10% of haematological cancers. The introduction of novel drugs, such as proteasome inhibitors and various immunotherapies, has increased the survival time for MM patients and changed the view of MM to a chronic manageable disease [24]. MM is a heterogenic disease featuring several different characteristics including altered signalling and increased metabolic activity [6,25]. In this study, we examined the PCNA-dependent regulation of metabolism and signalling further in seven MM cells with primary cell-like properties. We examined the correlation between gene expression and activation of signalling proteins in untreated cells and found a correlation between increased ribosomal gene expression, and thus ribosomal activity, and increased proteasomal and ER stress. Interestingly, this was also associated with increased sensitivity to ATX-101 and a metabolic shift upon ATX-101 treatment. Protein pull-down of activated signalling proteins identified 11 proteins that were found only in ATX-101-sensitive MM cell lines, of which 5 have previously been implicated with ER stress and/or redox balance. These proteins could therefore be biomarkers of increased ER stress, as well as sensitivity to ATX-101 treatment. ATX-101 showed additive effects with proteasome inhibitors, and proteasome inhibitor-resistant MM cells were hypersensitive to ATX-101. These results indicate that PCNA is a promising drug target in subgroups of MM, alone or in combination with proteasome inhibition.

## 2. Materials and Methods

### 2.1. Drugs and Inhibitors

These included APIM-peptide, ATX-101 (Ac-MDRWLVKWKKKRKI-RRRRRRRRRRR) (APIM Therapeutics, Rissa, Norway) and ATX-A (Innovagen, Lund, Sweden), described in [3,13], melphalan (Sigma-Aldrich, Burlington, MA, USA), carfilzomib (CFZ) (Selleck Chemicals, Houston, TX, USA) and ebselen (Sigma-Aldrich).

### 2.2. Cell Lines

The JJN3 cells were as described elsewhere [13]; the WT and carfilzomib-resistant AMO1 cell line (AMO1-Carf) was a kind gift of Professor Christopher Driessen (Kantonsspital St. Gallen, St. Gallen, Switzerland) and was generated as previously described [26]; and the TK cell lines were isolated from peripheral blood (PB), bone marrow (BM) or a pleural effusion in the pleural cavity of the lungs (PE) from five MM patients (Table 1) and are from Professor Andrew Spencer’s laboratory. These established cell lines do not express the Epstein–Barr virus nuclear antigen. Below is a table outlining the cytogenetics and prior treatments.

Because some of the cell lines were semi-adherent, TK9, 12, 13 and 18 were verified to be MM cells by flow cytometry using anti-CD138 antibody (Abcam, Cambridge, UK, ab128936).

### 2.3. Cell Line Culture Conditions

All cell lines were cultured in RPMI 1640 (Sigma Aldrich) supplemented with 2 mM glutamine (Biochrome, Waterbeach, UK), 100 µg/mL gentamicin (Sigma-Aldrich) (except for AMO-1 cells), 2.5 µg/mL amphotericin (Sigma-Aldrich) and 10% foetal bovine serum (Sigma-Aldrich) and maintained at 37 °C in a humidified atmosphere of 5% CO_2_. All TK cell lines were derived from relapsed refractory multiple myeloma patients following written informed consent with approval from the Alfred Hospital Research and Ethics Committee.

### 2.4. Analysis of mRNA Sequences

Total RNA from the TK cell lines was extracted in triplicate from 3 consecutive cell line passages and subjected to eukaryotic RNA-seq (mRNA-enriched method) and sequenced 20M reads on HiSeq, PE150 mRNA sequencing by Novogene Bioinformatics Technology Co., Ltd. Sequencing data were quality-checked using nf-core framework (ref https://www.nature.com/articles/nbt.3820, accessed on 2 December 2023); code and results available at https://github.com/animesh/spritz_nf (accessed on 2 December 2023). It was analysed using the https://github.com/nf-core/rnaseq/releases/tag/3.13.2 pipeline (accessed on 2 December 2023). The counts from the technical replicate were summed up using custom base-R version 4.3.2 (R: The R Project for Statistical Computing (r-project.org)), script: https://raw.githubusercontent.com/animesh/scripts/1863c57d212b8b993a367bbe0d92255b5ab5a159/diffExprSeq.r (accessed on 5 February 2023). The result was further processed with differential abundance pipeline https://github.com/nf-core/differentialabundance/releases/tag/1.3.1 (accessed on 5 February 2023). These pipelines were executed on high-performance supercomputing infrastructure Saga (https://documentation.sigma2.no/hpc_machines/saga.html, accessed on 2. December 2023) provided by Sigma2—the National Infrastructure for High-Performance Computing and Data Storage in Norway, where slurm processes were orchestrated via https://tower.nf/orgs/NTNU/workspaces/TK/watch/5xEXDjE3TFgJV0 (accessed on 2 December 2023) in order to be reproducible following FAIR [27] guidelines. The data are deposited in The Sequence Read Archive (BioProject ID PRJNA1176350 http://www.ncbi.nlm.nih.gov/bioproject/1176350, accessed on 23 October 2024).

### 2.5. Mass Spectrometric Metabolite Profiling

TK cell lines were seeded in ultra-low-attachment surface-coated culture flasks (Corning Inc., Corning, NY, USA), incubated overnight to reach a cell density of 3–4 × 10^5^ cells/mL and treated with ATX-101 (10 µM), Melphalan (1 µM) or the respective combination for 4 h. Three replicate cultures (n) were sampled by fast filtration and prepared for downstream MS analysis as described in [6,28]. Phosphorylated metabolites and tricarboxylic acid intermediates were measured by capillary ion chromatography (capIC) tandem mass spectrometry (MS/MS), while lactate, pyruvate and amino acids were quantified by two liquid chromatography (LC) MS/MS methods, all as described for haematological cell lines in [6,28]. Extract concentrations were normalised to total protein (mg/mL) of the respective culture.

### 2.6. Multiplexed Inhibitor Bead (MIB)-Assay

An assay of non-targeted enrichment of activated (ATP/GTP) binding proteins, or proteins in complex with these, originally described by Duncan and colleagues [29], was performed as previously described [6,29].

### 2.7. Mass Spectrometry Data Analysis

Cell extracts for the MIB assay (kinase enrichment) were prepared as described [30]. Proteins were quantified by processing MS data using MaxQuant v.1.6.10.43 [31]. Open workflow [32] was used to inspect the raw files to determine optimal search criteria. Namely, the following search parameters were used: enzyme specified as trypsin with a maximum of two missed cleavages allowed; acetylation of protein N-terminal, oxidation of methionine, deamidation of asparagine/glutamine and phosphorylation of serine/threonine/tyrosine as dynamic post-translational modification. These were imported in MaxQuant, which uses *m*/*z* and retention time (RT) values to align each run against each other sample with a minute window match-between-run function and 20 min overall sliding window using a clustering-based technique. These were further queried against the human proteome including isoforms downloaded from Uniprot (https://www.uniprot.org/proteomes/UP000005640, in 1 September 2019) and MaxQuant’s internal contaminants database using Andromeda built into MaxQuant. Both protein and peptide identification false-discovery rates (FDRs) were set to 1%; only unique peptides with high confidence were used for final protein group identification. Peak abundances were extracted by integrating the area under the peak curve. Each protein group abundance was normalised by the total abundance of all identified peptides for each run and protein by calculated median summing all unique and razor peptide-ion abundances for each protein using a label-free quantification (LFQ) algorithm [33] with minimum peptides ≥ 1. LFQ values for all samples were combined and log-transformed with base 2, and the transformed control values were subtracted. The resulting values reflecting the change relative to control for each condition were subjected to a two-sided non-parametric Wilcoxon sign rank test [34] as implemented in MATLAB R2020a (Math Works Inc., Natick, MA, USA, https://www.mathworks.com/) in order to check the consistency in the directionality of the change, namely, a negative sign reflecting the decreased and positive sign reflecting the increased expression of the respective protein group. The choice of this non-parametric test avoids the assumption of a certain type of null distribution as in the Student’s *t*-test by working over the rank of the observation instead of the observation value itself. Further, it also makes it robust to outliers and extreme variations noticed in observed values. Differentially expressed (DE) protein groups were identified at p 0.25. The Uniprot accession IDs of these DEs were mapped to pathways (https://wikipathways-data.wmcloud.org/current/gmt/, accessed on 30 October 2020) using R (https://www.R-project.org/) libraries, org.Hs.eg.db and clusterProfiler [35]. Venn diagrams were built using the R package limma [36] and heat maps using pheatmap (https://academic.oup.com/nar/article/43/7/e47/2414268, accessed on 30 October 2020). Online Ingenuity^®^ Pathway Analysis™ software (QIAGEN Inc., Venlo, The Netherlands, https://digitalinsights.qiagen.com/products-overview/discovery-insights-portfolio/analysis-and-visualization/qiagen-ipa/, accessed on 30 October 2020) was used to combine with metabolomics data for annotation, visualization and integrated discovery of canonical pathways and other functional analysis. The proteomic data for the TK cell lines are deposited in PRIDE [37], project IDs PXD033510 (https://www.ebi.ac.uk/pride/archive/projects/PXD033510/, TK12, 13, 14 and 16) and PDX033531 (https://www.ebi.ac.uk/pride/archive/projects/PXD033531, TK9), both accessed on 28 April 2022. Raw data from the MM cell line JJN3, the B lymphoblastoid cell line MCCAR, the acute myeloid leukaemia cell lines NB4 and HL60 and primary human monocytes from three donors (also described in [6]) have project IDs PXD028314, PXD017474; from the bladder cancer cell lines UmUc-3 and T24 (also described in [5]) PXD011044; the sarcoma cell line U2OS and the two lung cancer cell lines H460 (non-small cell lung cancer, NSCLC) and A549 (squamous cell lung cancer) (also described in [30]) PXD005286.

### 2.8. Whole Genome Gene Expression Analysis

Total RNA was isolated from snap-frozen pellets by a RNeasy kit (Quiagen). Genome-wide gene expression profiling analysis was performed as described [38]. The microarray experiments are MIAME (Minimum Information About a Microarray Experiment)-compliant and were deposited in the ArrayExpress database (http://www.ebi.ac.uk/arrayexpress/, accessed on 23 October 2024) under accession number E-MTAB-5644. Gene expression raw data were normalised and analysed using GeneSpring 12.6—GX, probes were filtered by Flags and fold change ≥1.1. Similar up- and downregulated genes from duplicate/triplicate experiments were extracted.

### 2.9. HIF1A Levels

Whole-protein extracts (triplicates) were prepared from JJN3 pellets (4 °C, 150× *g*, 5 min) according to the cell extraction protocol for ELISA sample preparation by ThermoFisher Scientific. HIF1A levels were measured in duplicate, applying an HIF1A Human ELISA kit (Invitrogen, Waltham, MA, USA) according to the manufacturer’s instructions. Measured HIF1A levels were normalised to cell density to obtain the amount of HIF1A (fg)/cell.

### 2.10. Viability Assays

Cell viability was assessed by an MTT assay, as described in [8], or by a PrestoBlue viability assay as previously described [6]. JJN3 cells were seeded at 5 × 10^4^ cells/mL, incubated in a humidified atmosphere of 5% CO_2_ and either atmospheric O_2_-levels (normoxia) or 1% O_2_ (hypoxia), the latter in a C-chamber incubator subchamber controlled by a ProOx C21 compact O_2_ and CO_2_ subchamber controller from BioSpherix. The AMO-1 cell lines were seeded at a density of 5 × 10^4^ cells/mL. The cells were treated with ATX-101 (2–12 µM) and carfilzomib (CFZ) (0.032–2500 nM) before being incubated in a humidified atmosphere of 5% CO_2_ for 1–4 days. DMSO in the same concentration as used for the highest CFZ concentration was used as CFZ control. It did not affect the fluorescence compared to untreated control. The TK cell lines were seeded at 3–5 × 10^4^ cells/mL and treated with ATX-101 (4 and 8 µM), melphalan (1 µM) or the respective combinations for 1–4 days.

### 2.11. LC-MS/MS Analysis of Pyridine Nucleotides

Intracellular NAD+ and NADH was extracted from JJN3, RPMI8226, HL60, HEK293 and DU145 pellets (4 °C, 150× *g*, 3 min) by shaking (2 min, 80 °C, 1500 rpm) in 300 µL water–acetonitrile with 10 mM acetic acid, adjusted to pH 9.0 with ammonium hydroxide (80 °C, 10–90 *v*/*v* %). Residual lipid and proteins were cleared from the extracts by spin filtration in 3-kDa-molecular-weight spin cut-off filter (VWR) and 3 mg C18-material (Waters) was added. NAD and NADH levels were quantified on an ACQUITY I-Class UPLC/Xevo TQ-S triple quadrupole MS system (Waters) operated in negative electrospray mode. Samples (5 µL) were injected onto a Waters ACQUITY UPLC BEH-Amide 1.7 µm 2.1 × 100 mm column, maintained at 40 °C and eluted with mobile phases (A) water–acetonitrile (60–40 *v*/*v*), and (B) water–acetonitrile (10–90 *v*/*v*), both with 10 mM acetic acid added and adjusted to pH 9.0 with ammonium hydroxide. The following gradient was applied with a flow rate of 0.4 mL/min: 0–0.5 min: 95% B, 0.5–2 min: 95–70% B, 2–6.5 min: 70–40% B, 6.5–7 min: 40–1% B, 7–8 min: 1% B, 11 min: end. Analytical grade standards (Sigma-Aldrich) were prepared fresh in mobile phase B. Both analytical standards and extracts were analysed within four hours of preparation. Intracellular NAD+, NADH, NADP+ and NADPH in TK cell lines were extracted, analysed and quantified from pelleted cells as described in [39].

### 2.12. GSH-ELISA

GSH levels were measured using the GSH-GloTM Glutathione Assay (Promega) according to the manufacturer’s instructions.

### 2.13. LC-MS/MS Analysis of GSH and GSSG

Intracellular glutathione was extracted, derivatized and quantified by a protocol modified from [40,41,42,43]. Pelleted TK cells (4 °C, 150× *g*, 3 min) were re-suspended in 1.5 mL phosphate buffer saline (PBS, Sigma-Aldrich). An amount of 100 µL of diluted (1:20) cell suspension was next incubated (5 min, 300 rpm) with 1.2 µL N-ethylmaleimide (NEM, 310 mM, Sigma-Aldrich) for derivatization and extracted and deproteinized by vortexing with 300 µL ice-cold methanol (Sigma-Aldrich), then 10 µL internal standard (5 µM GS-NEM-13C2,15N, derivatized from glutathione–(glycine-13C2,15N), Sigma-Aldrich) was added. The extracts were incubated on ice (10 min) with occasional vortexing and cleared by centrifugation (4 °C, 10 min, 14,000 rpm). An amount of 350 µL of extract supernatant was evaporated under nitrogen gas and reconstituted in 100 µL LC-MS-grade water for downstream analysis. GS-NEM and GSSG extract levels were quantified on an ACQUITY UPLC I-Class UPLC coupled to a Xevo TQ-XS triple quadrupole mass spectrometer (Waters) operated in positive electrospray mode as described by [41] with slight modifications. Next, 5 µL samples were injected onto an ACQUITY UPLC HSS T3 Column (1.8 µm, 2.1 mm × 150 mm) maintained at 40 °C and eluted with mobile phases (A) water and (B) acetonitrile, both with 0.1% formic acid (*v*/*v* %) added. The following gradient was applied with a flow rate of 0.4 mL/min: 0–2 min, 0.2–40% B; 2–3.5 min, 40–70% B; 3.5–3.6 min, 70–99% B; 3.6–4.5 min, 99% B; 4.5–4.6 min, 99–0.2% B; 4.6–6.5 min, 0.2% B. The instrument was operated at a capillary voltage of 3.0 kV, a source temperature of 150 °C, a desolvation gas flow of 900 L/h and a desolvation temperature of 350 °C. Quantification was performed from the following precursor-product ion transitions: GS-NEM: *m*/*z* 433 > 304 and GSSG *m*/*z* 613 > 355, normalised to GS-NEM-13C2,15N *m*/*z* 436 > 307. Extract concentrations were interpolated from a 16-point calibration curve prepared from serial dilutions of analytical-grade standards treated as the cell extracts and calculated by least squares regression. Extract concentrations were normalised to total protein (mg/mL) of the respective culture.

### 2.14. Determination of Total Protein for Normalisation

Cells were pelleted, washed in phosphate-buffered saline (150× *g*, 5 min, 4 °C) and re-suspended in 3× packed cell volume of M-PER protein extraction reagent (Thermo Fisher Scientific, Waltham, MA, USA) substituted with 10 µL/mL Halt Protease and Phosphatase Inhibitor Cocktail (Thermo Fisher Scientific), 1 mM dithiothreitol (Sigma-Aldrich) and 200 units OmniCleave Endonuclease (Lucigen Corporation, Middleton, WI, USA) in Protein LoBind tubes (Eppendorf, Hamburg, Germany). Cells were extracted on ice for 1.5 h with occasional vortexing, and extracts were cleared by centrifugation (14,000× *g*, 15 min, 4 °C). Extract absorbance was measured at 280 nm on a NanoDrop One Spectrophotometer with a baseline correction wavelength of 340 nm. Absorbance was corrected for nucleic acid impurities at 280 nM to allow for calculation of corrected total protein concentration assuming 1 Abs = 1 mg/mL.

### 2.15. 6PGD Activity Assay

6PGD activity was measured in TK16 cells left untreated or treated with ATX-A (10 μM), ATX-101 (10 μM) or Ebselen (30 μM) for 4 h using a 6-phosphogluconate dehydrogenase assay kit (Abcam, ab241016). In this assay, 6PGD catalyses the conversion of 6-phosphogluconate in a reaction generating NADPH that subsequently reduces a colourless probe to a coloured product that can be read at absorbance 460 nm. Thus, the higher the absorbance, the higher the 6PGD activity. The assay was conducted as described by the supplier. In brief, cells were harvested and lysed in 6PGD assay buffer and the lysates collected. 6PGD developer and 6PGD substrate were added to the lysates in 96-well plates and the absorbance measured (OD460 nm, 45 min, 37 °C).

### 2.16. Western Blot

TK cell lines were seeded in ultra-low-attachment surface-coated culture flasks (Corning) and incubated overnight to reach a cell density of 3–4 × 10^5^ cells/mL prior to treatment with ATX-101 (10 µM) or ATX-A (10 μM) for 4 or 24 h. Cells were harvested by centrifugation and lysed in M-PER buffer (3× packed cell volume, Thermo Fischer Scientific) with Halt Protease & Phosphatase inhibitor cocktail (1×, Thermo Fischer Scientific), DTT (1 mM) and Omicleave (1 µL, Lucigen) added and then incubated on ice for 1.5 h with vortexing every 30 min. The supernatant was collected as total cell extract. DTT (0.1 M) and LDS loading buffer (1×) were added to the cell extracts (50 μg), then incubated for 10 min (70 °C) prior to separation by gel electrophoresis (4–12% Bis-Tris gels, NuPAGE, Invitrogen) and blotted to polyvinylidene fluoride membranes (0.2 μM, Immobilon, Merch Millipore, Burlington, MA, USA) or nitrocellulose membranes (0.2 μM, Invitrogen). Membranes were blocked in dry milk (5% in TBS) and incubated overnight with primary antibodies against ENO1 (Abcam, ab227978), 6PGD (Santa Cruz Biotechnology, sc-398977), GAPDH (Abcam, ab8245), PCNA (PC-10, Santa Cruz Biotechnology, sc-56), AKT (Cell signalling technologies (CST), #4691), AKT-P (CST, #4060). H3 (Abcam, ab1791) and EIF2S1 (Sigma Aldrich, HPA 064885) were used as loading controls because these proteins showed bands on the Western blots that corresponded to the number of proteins added to the wells in all cell lines, while β-tubulin and β-actin did not. The fluorescently labelled secondary antibodies IRdye 680RD goat α-mouse (LI-COR) and IRdye 800CW goat α-rabbit (LI-COR) were used for protein detection. Proteins were visualized by an Odyssey infrared imaging system (LI-COR Biosciences) and quantified in Odyssey Image Studio (V2.0). Protein levels were normalised to H3 and EIF2SL for loading and levels and presented either as relative to levels in untreated cells or as relative to levels in the TK18 cell line.

## 3. Results and Discussion

### 3.1. Metabolome, Proteome and Transcriptome Profiling Support a Role of PCNA in Regulation of Primary Metabolism, Immune Response and Redox Capacity in JJN3 Cells

**Figure 1:**
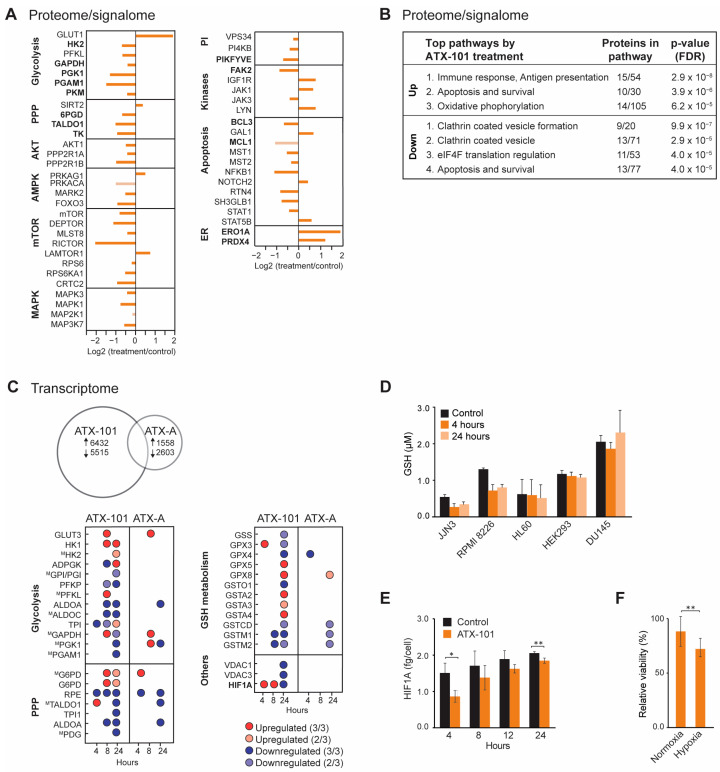
Systemic changes after ATX-101 treatment of MM cells lead to reduced primary metabolism, increased ER stress and reduced redox capacity. (**A**) Log2 fold change in proteins detected by MIB assay performed on extract from JJN3 cells treated with ATX-101 (6 µM) for 4 h. Proteins in bold are mentioned specifically in the text. Data shown are mean of three repeated experiments relative to untreated control. Dark-coloured bars indicate significant change from untreated control (Wilcoxon sign rank test). (**B**) Top up- and downregulated STRING GO (KEGG) pathways based on significantly changed proteins in the MIB assays according to the Wilcoxon sign rank test. (**C**) Number of DE genes (upper panel) and up/downregulated DE genes in specific pathways (lower panel) in JJN3 cells after 24 h of APIM- or ATX-A (6 µM) treatment. M denotes that the gene product also was detected by the MIB assay. Average of three repeated experiments for 24 h and two repeated experiments for 4 and 8 h (included only if same trend in both) is shown. Dark-coloured circles are DE genes in three out of three replicates, while light-coloured circles are DE genes in two out of three replicates. (**D**) Average GSH levels after ATX-101 (6 µM: JJN3, RPMI 8226, HL60 and DU145 or 8 µM: HEK293) treatment for 4 and 24 h. Data based on triplicates from two (JJN3, HEK293 and RPMI 8226) or three (DU145 and HL60) repeated experiments. (**E**) Average HIF1A protein levels (fg/cell) in JJN3 cells 4, 8, 12 and 24 h after treatment with ATX-101 (8 µM) ± SD, *n* = 3. (**F**) Average viability relative to untreated control cells measured by the MTT assay in JJN3 cells treated with ATX-101 (6 µM) under atmospheric O_2_ tension (normoxia) or 1% O_2_ (hypoxia) at 24 h ± SD, *n* = 10. Data from one representative out of two repeated experiments are shown. * *p* ≤ 0.5, ** *p* ≤ 0.01, *t*-test. These results were previously published in [48].

Because reduced glucose uptake and a clear metabolic shift with reduced metabolite pools in nucleoside metabolism, glycolysis and PPP were detected after ATX-101 treatment in several haematological cells [6], the MM cell line JJN3 was used as a model to further explore the cellular effects upon ATX-101 treatment, including changes in cellular signalling, transcriptome and redox balance. The MIB assay, which is a non-biased untargeted mass spectrometry (MS)-based assay based on the pull-down of activated signalling proteins (GTP/ATP binders) and/or proteins in complex with these via kinase inhibitors, i.e., the active signallome, was here used to examine changes in cellular signalling [6,29,30]. Using this assay, we pulled down less 6PGD from ATX-101-treated JJN3 cells (Figure 1A, PPP), in agreement with previous published data showing a reduction in 6PGD protein levels (using Western analysis) as well as in 6PGD activity upon ATX-101 treatment in several cells [6]. Reduced pull-down of transaldolase (TALDO1) and transketolase (TK) further supports reduced PPP, and reduced pull-down of multiple glycolytic proteins including hexokinase 2 (HK2), GAPDH, phosphoglycerate kinase 1 (PGK1), PGAM1 and PKM supports perturbed glycolysis in agreement with previous published reduced metabolism upon ATX-101 treatment [6] (Figure 1A). Of these proteins, GAPDH and PKM are reported to be stabilised by and/or in complex with PCNA [6,19].

Treatment with ATX-101 reduced pull-down of several signalling proteins important for the regulation of metabolism, e.g., proteins in the AKT, AMPK, mTOR and MAPK pathways (Figure 1A). Out of the other signalling proteins, the most evident changes were the reduction of several phosphatidylinositol (PI) kinases including PIKFYVE, which is also important for vesicular transport, and the non-receptor tyrosine kinase FAK2, which is known to activate MAPK and PI3K/AKT signalling and to communicate with ENO1 in glycolysis [44]. FAK2 was previously also identified in PCNA complexes [3], further supporting a scaffold role for PCNA in cellular signalling. Several proteins regulating apoptosis were reduced, for example, B-cell lymphoma 3-encoded protein (BCL3) and myeloid cell leukaemia 1 (MCL1), both proteins associated with increased proliferation and malignancy in MM [45,46]. While the pull-down of many proteins was reduced upon ATX-101 treatment, two ER proteins, peroxiredoxin 4 (PRDX4) and endoplasmic reticulum oxidoreductase 1 alpha (ERO1A), were strongly increased (Figure 1A). This suggest that ATX-101 treatment increases the cellular ER stress. Increased pull-down of proteins involved in oxidative phosphorylation, and of some DNA repair and replication proteins, was also detected (Supplementary Figure S1A). Increased respiratory activity upon reduced glycolysis was previously detected in cells where ENO1’s ability to bind to PCNA is impaired [6]. Taken together, these changes show that targeting the scaffold function of PCNA affects several stress responses and signalling networks in JJN3 (summarized in Supplementary Figure S2).

STRING GO pathway analysis of changed proteins after ATX-101 treatment (Figure 1B) pointed to alterations in pathways regulating immune responses, apoptosis, oxidative phosphorylation, translational regulation and vesicle formation. Impaired vesicle formation and function is expected to lead to an increase in pull-down of membrane proteins due to increased concentrations of these proteins in the cell extracts, and this is what we detected in our dataset, as ~80% of the upregulated proteins belonged to a list of membrane proteins, the “membranome” [47] (Supplementary Figure S1B).

Next, transcriptome profiling was performed, and we included a mock peptide, a mutated ATX-101 (ATX-A, W2A mutation in the APIM motif) with lower affinity for PCNA [3]. ATX-101 treatment led to a much higher number of differentially expressed (DE) genes compared to ATX-A (Figure 1C). We also detected more DE genes involved in glycolysis, PPP and GSH metabolism after ATX-101 than ATX-A treatment, supporting that the alterations in these pathways were linked to the ability to interact with PCNA. While large changes in the metabolome [6] and proteome (Figure 1A, B) were evident after 4 h, the changes in gene expression were more prominent after 24 h (Figure 1C). Most of the DE genes in glycolysis and PPP were downregulated 24 h after ATX-101 treatment.

GSH/GSSG (reduced/oxidised glutathione) is important in the cellular defence against oxidative stress, for example, caused by ER stress which seems to be increased by ATX-101 treatment (Figure 1A). Accordingly, the two MM cell lines JJN3 and RPMI 8226 responded to ATX-101 treatment with lowering their GSH levels (Figure 1D). This was not seen in the three non-MM cell lines examined. These data support that the redox balance is affected by ATX-101 as indicated by the transcriptome results.

HIF1A is a transcription factor known to upregulate glycolysis and to be a mediator of the cell’s response to hypoxic conditions, a condition often found in, for example, tumours in the bone marrow. The main regulation of the HIF1A protein levels is via von Hippel–Lindau (VHL)-mediated ubiquitination and degradation of hydroxylated HIF1A (in presence of oxygen), while gene expression is regulated via, for example, the PI3K/AKT pathway [49]. ATX-101 reduces the glycolysis, and the mRNA expression level of HIF1A was increased at 4 and 8 h, while it was reduced after 24 h (Figure 1C). However, at the protein level, HIF1A was reduced at both 4 and 24 h (Figure 1E). These results suggest that ATX-101 reduced HIF1A levels both on the protein and transcriptome levels and that these responses are separated in time. In support of an ability of ATX-101 to perturb a normal HIF1A response, we found that ATX-101 treatment had a stronger growth inhibitory effect on JJN3 cells under hypoxic, i.e., when HIF1A normally should have been increased, than under normoxic conditions (Figure 1F). How ATX-101 affects HIF1A levels is elusive, but it could be associated with reduced PI3K/AKT signalling (here, [3,5,6,14]).

### 3.2. Sensitivity to Targeting PCNA with ATX-101 Differs Between Several MM Cell Lines but Correlates with Increased Translation

**Figure 2:**
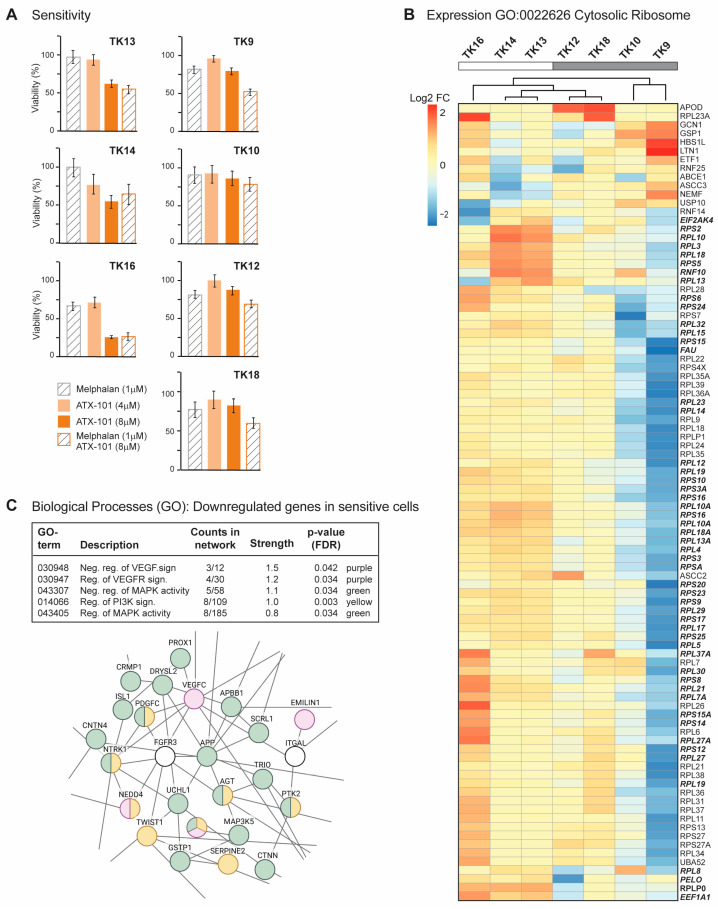
Sensitive TK cells have elevated expression of genes involved in translation (**A**) Viability measured by the MTT assay relative to untreated control presented for TK9, 10, 12, 13, 14, 16 and 18 cell lines on day 4 after treatment with ATX-101 (4 or 8 µM), melphalan (1 µM) and the combination of ATX-101 (8 µM) and melphalan. The average of 6 replicate wells from one representative out of two repeated experiments is shown. (**B**) Expression of genes in GO:0022626, cytosolic ribosome and cluster analysis for TK9, 10, 12, 13, 14, 16 and 18. Bolded italic genes have increased expression in TK13 and TK14 compared to TK12. (**C**) Selected biological processes (GO) of genes downregulated in sensitive (TK13, 14 and 16) relative to not sensitive (TK9, 10, 12 and 18) cells. GO network analysis revealed networks with 153 nodes/126 edges and a PPI enrichment *p*-value of 1.08 × 10^–9^. A subnetwork where proteins in VEGF (purple)/MAPK (green) and PI3K (yellow) signalling pathways is shown.

MM is a heterogeneous cancer, and commonly used commercial cell lines such as JJN3 and RPMI 8226 can differ significantly from primary cells. A panel of seven newly established MM cell lines, known as the TK cell line (Table 1), was therefore next investigated. Due to their low passage numbers, these cell lines are likely to have more primary cell-like properties than the commercial MM cell lines, and they all grow slower than JJN3. TK 9, 10 and 14 grew slower than TK12, 13, 16 and 18, which had approximately the same growth rate. As TK13, 14 and 16 were found to have higher sensitivity to ATX-101, the sensitivity is not linked to growth rate (Figure 2A, left panel). ATX-101 reduced the viability in these cells compared to the untreated control by 40–75% on day 4, which is similar to what is seen in the commercial cell lines JJN3 and RPMI 8226 [13] (IC50 data for all cell lines used in this paper are shown in Supplementary Table S1).

Melphalan is an alkylating chemotherapeutic agent used for treatment of MM. Previously, an additive effect of ATX-101 and melphalan was demonstrated in JJN3 and RPMI 8226 [13]. This is thought to be due, at least in part, to an increased affinity of the APIM motif, and thus ATX-101, for PCNA during cellular stress, resulting in increased impairment of PCNA’s scaffold roles [6,8,13,14,15]. The cytotoxic effects of melphalan were low in all TK cell lines (Figure 2A), and melphalan did not increase the effect of ATX-101 in the sensitive cell lines. However, the combinations of ATX-101 and melphalan was more potent than either single treatment in the less sensitive cell lines, TK9, 10, 12 and 18, suggesting that increased stress enhances the effect of ATX-101 in these cells. Sensitivity to ATX-101 was not correlated with the growth rates of the cell lines nor with the site from which they were isolated (Table 1). Three of the cell lines were isolated from the same patient at different stages of the disease (TK12, 13 and 14), and the sensitivity to ATX-101 increased as the disease progressed.

Analyses of the transcript expression profile of the seven TK cell lines revealed that TK16 is very different from the other sensitive cells (MAD score ~−2.8,). Nevertheless, TK16 was included in the ATX-101-sensitive group together with TK13 and TK14. The genes with the largest variation in expression (both decreased and increased based on log counts) in this group were compared to the less sensitive group (TK9, 10, 12, 18) and analysed for GO terms. No GO terms were found to be significantly enriched when looking at the genes with higher expression in the sensitive compared to the less sensitive group (FDR 0.1). The most upregulated single gene was prostaglandin F2 receptor negative regulator (PTGFRN, log2FC~10), a protein shown to be upregulated and correlated with poor survival in glioblastoma multiforme [50], and the gene with highest significance was an LncRNA (ENSG00000266976) on chr 19 with unknown function. By presenting the list of the genes, ranked from low to high adjusted *p*-values to STRING [51], we found that many of the high-ranked genes belong to the GO term cytosolic ribosome (GO:0022626, enrichment score of 96 out of 101 proteins, FDR 1.5 × 10^−6^). We next performed a cluster analysis of these genes and found that TK13, 14 and 16 cluster together and that these sensitive cell lines generally have a higher expression of these genes than the other cell lines (Figure 2B). Note that TK12, from the same patient as TK13 and 14, clusters closest to TK13. We also see a clear increase in expression from TK12 to TK13 and/or TK14 for many of these genes (Figure 2B, bold italics). This may indicate that increased gene expression of ribosomal genes follows disease progression.

Several significant GO processes were found to be enriched for the group of genes that were less expressed in ATX-101-sensitive cells (TK13, 14 and 16) compared to the other cell lines (TK9, 10, 12 and 18). These processes include VEGF, MAPK and PI3K signalling pathways (Figure 2C, upper panel). A part of the total network containing 153 nodes/126 edges and a PPI enrichment *p*-value of 1.08 × 10^−9^, is shown in Figure 2C. This subnetwork includes multiple genes encoding proteins involved in immune responses (e.g., DAB2IP, TWIST1), ubiquitin processes (e.g., NEDD4, UCHL1) and redox processes (e.g., GSTP1).

### 3.3. Sensitivity to Targeting PCNA with ATX-101 Correlates with Elevated ER Stress

**Figure 3:**
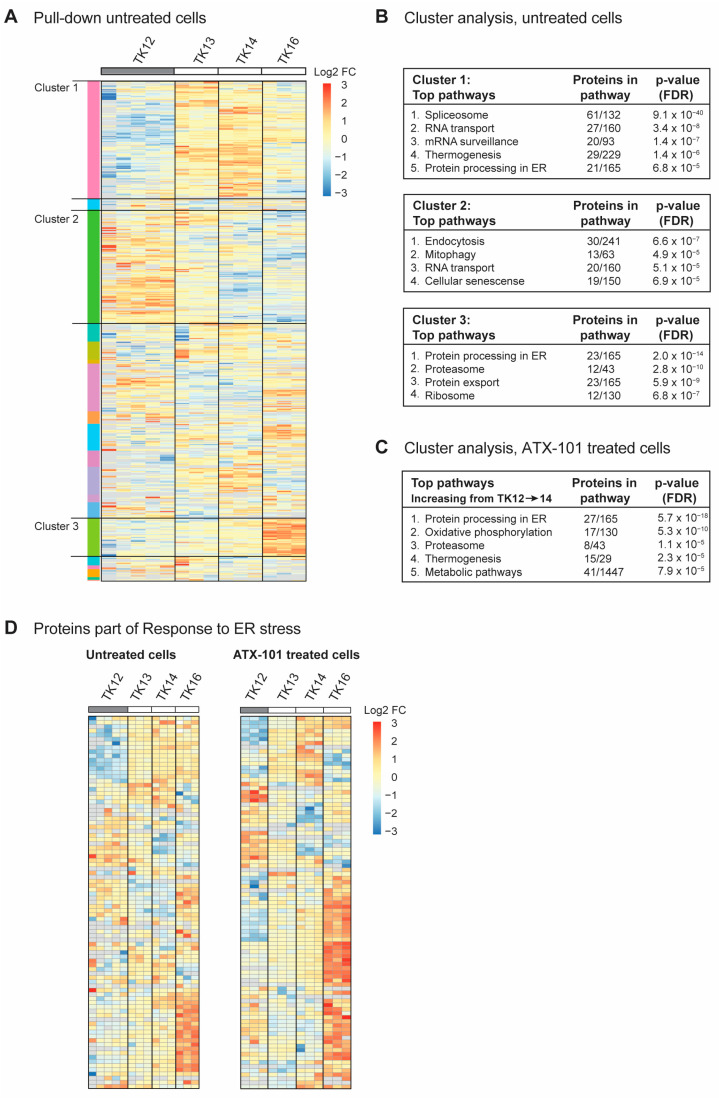
Sensitivity to ATX-101 increases with increasing ER stress. (**A**) Hierarchical clustering of all quantified protein groups in controls (untreated) TK12, 13, 14 and 16 cells. Cell line over grey box is less sensitive to ATX-101. (**B**) Top KEGG pathways enriched in clusters 1, 2 and 3 shown in (**A**), identified by STRING GO analysis. (**C**) Top KEGG pathways found in the only cluster (same analysis as in (**A**)) with increasing pull-down of proteins from TK12-16- in ATX-101-treated cells. (**D**) Cluster analysis of all proteins in untreated and ATX-101-treated cells belonging to GO:003476, response to ER stress. Same heat maps with protein identifications are shown in Supplementary Figure S3. Data from 3–5 independent biological replica are shown.

To investigate whether there were any trends in the signallome that could explain why the sensitivity to ATX-101 increased during disease progression in the same patient (TK12, 13 and 14), all proteins pulled down from untreated cells using the MIB assay were subjected to a cluster analysis. We also included data from the most sensitive cell line (TK16), as changes related to ATX-101 sensitivity were of the most interest. Increased pull-down of proteins in the MIB assay suggests that the proteins are increasingly activated, whereas decreased pull-down suggests that the proteins are either less activated or degraded. Hierarchical clustering of all quantified protein groups in untreated cells, excluding those marked as contaminants/reverse hits (3936 out of 4062), using the values normalised by median expression in log2 space with Pearson correlation and average linkage, yielded 20 clusters, of which 3 (designated clusters 1–3) covered >50% of all the proteins. Cluster 1 showed an increasing protein pull-down from TK12 to TK14 (Figure 3A), and STRING GO analysis of this cluster indicated that pathways (KEGG) involved in splicing, RNA transport and surveillance, thermogenesis and ER processes were activated during disease progression (Figure 3B, upper panel). TK16, the most sensitive cell line, did not follow the same trends for most of the proteins in this cluster. Cluster 2, on the other hand, showed a trend of decreasing pull-down from TK12-16, i.e., also reduced in the most sensitive TK16 cell line (Figure 3A). These proteins belong to pathways regulating endocytosis, mitophagy, RNA transport and cellular senescence (Figure 3B, middle panel). Cluster 3 (Figure 3A) showed an increasing trend in the pull-down from TK12-16, suggesting an increased activation of these proteins in all the sensitive cells. These are proteins in ER processing, proteasome, protein export, and ribosomes (Figure 3C, bottom panel). The latter group was also found to be increased at the gene expression level (Figure 2B); however, we did not see an increase in the expression of genes related to ER stress in the sensitive cells compared to the non-sensitive cells. Therefore, the increased pull-down of ER stress proteins using the MIB assay is likely related to protein activation rather than protein levels. However, increased expression of ribosomal genes and activation of ribosomal proteins may indirectly lead to increased ER stress and increased proteasomal activity.

When all the proteins pulled down from ATX-101-treated cells were analysed in the same way as for untreated cells, we found only one cluster with a clear trend, i.e., an increase in the pull-down from TK12-16. STRING GO analysis of this cluster, which contained 340 proteins, showed that they belonged to pathways regulating ER processing, oxidative phosphorylation, proteasome, thermogenesis and metabolism (Figure 3C), i.e., pathways that were also found to be upregulated in untreated sensitive cells (Figure 3B, top and bottom panels).

Reduced mitophagy and increased protein processing in the ER suggests that the level of ER stress increases during disease progression (Figure 3B, top and middle panels, increasing or decreasing trends in TK12-14) and that this is important for sensitivity to ATX-101. Therefore, we next analysed the presence of proteins belonging to the GO group “Response to ER stress (GO:003476)”, which includes all the proteins involved in ER-associated degradation (ERAD) and unfolded protein response (UPR). Heat maps of the 91 proteins in this GO group from our data show that the pull-down of many of these proteins is higher in the more sensitive cell lines (TK13-16) and increases during disease progression, i.e., from TK12-14 (Figure 3D, same figures with protein identifications are shown in Supplementary Figure S3). The heat maps also show that ATX-101 further enhances the ER stress through, for example, increased pull-down of ERO1A (also increased in JJN3, Figure 1), eukaryotic translation initiation factor 2 alpha kinase 3 (EIF2AK3, also known as PERK), degradation in endoplasmic reticulum protein (DERL) 1 and 2 and thioredoxin-related transmembrane protein V (TMX) 3 and 4 (Supplementary Figure S3, bolded proteins). Activation of ER stress upon ATX-101 treatment has been previously demonstrated in breast cancer cell lines [14]; therefore, this effect of ATX-101 is not specific to MM cells. The increased oxidative phosphorylation detected in the ATX-101 treated cells (Figure 3C) is likely related to increased ER stress as this has previously been shown to activate mitochondrial activities including oxidative phosphorylation [52]. Increased oxidative phosphorylation was also detected in JJN3 cells upon ATX-101 treatment (Figure 1C, Supplementary Figure S1).

### 3.4. ATX-101 Further Increases ER Stress in Sensitive Cells While an Increase in Glycolysis and Central Signalling Pathways Is Detected in Less Sensitive Cells

**Figure 4:**
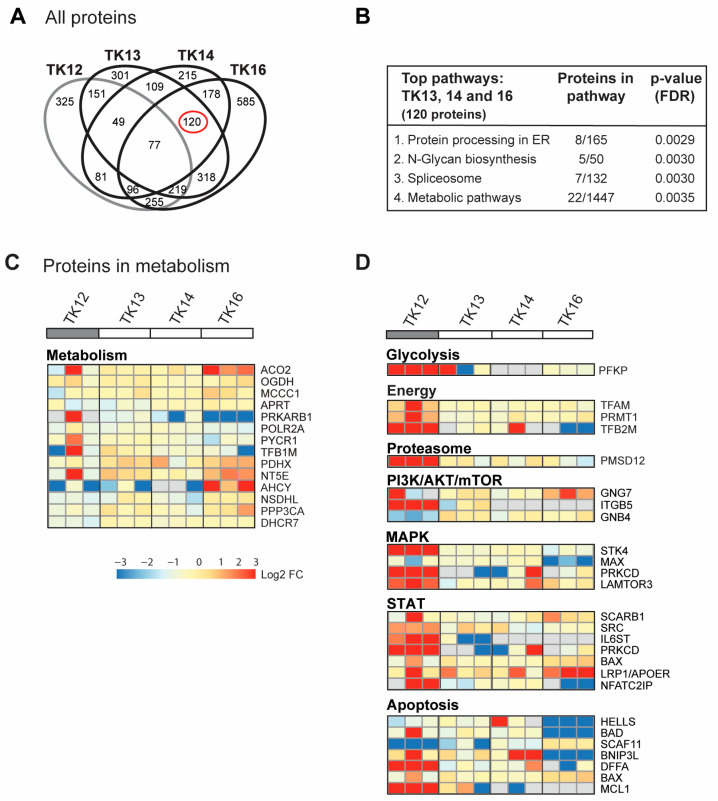
ATX-101 affects multiple signalling pathways differently in TK13, 14 and 16 versus TK12. (**A**) Venn diagram of proteins pulled down from ATX-101 (10 µM)-treated TK12, TK13, TK14 and TK16 cell extracts using MIB assay. Only proteins that are significantly changed from untreated control (changed in same direction in extracts from all three repeated experiments, Wilcoxon sign rank test) are shown. Full data set is deposit in PXD033510. (**B**) Enriched KEGG pathways (STRING) of proteins changed after ATX-101 treatment (10 µM) in in TK13, TK14 and TK16 (120 proteins) relative to untreated control. (**C**) Heat map of changes in proteins involved in metabolism (only including proteins significantly changed in more than 2 of the sensitive cell lines). Cell line over grey box is less sensitive to ATX-101. (**D**) Heat map of selected proteins involved in glycolysis, energy metabolism, proteasome, PI3K/AKT/mTOR, MAPK, STAT and apoptosis that changed in opposite directions in TK12 versus the sensitive cell lines (TK13, TK14 and TK16) (proteins significantly changed in more than 1 of the sensitive cell lines are included, for full pathway analysis see Supplementary Figure S4). Full data set is deposit in PXD033510.

In Figure 3D, all proteins in the GO group “response to ER stress” that changed after ATX-101 treatment in all three biological replicates of each cell line are included. Next, we analysed only the 900 proteins that changed in the same direction (up or down) in all three independent biological experiments (defined as significantly changed) following ATX-101 treatment compared to their untreated control. Although TK12, 13 and 14 were from the same patient, TK12 (the least sensitive) and TK16 (the most sensitive and from a different patient) have more changed proteins in common than any of the other cell line pairs. Changed levels of 77 proteins were detected in all cell lines after ATX-101 treatment, while 120 proteins were changed only in the sensitive cell lines TK13, 14 and 16 (Figure 4A, marked with red circle).

For the 77 proteins that changed upon ATX-101 treatment in all cell lines, no pathways were significantly enriched. However, the top four KEGG pathways enriched in the 120 proteins changed in the sensitive TK cell lines only, include protein processing in the ER and metabolic pathways (Figure 4B). In the analysis of proteins in metabolic pathways, when we relaxed the criteria slightly and included all proteins that were significantly changed in at least one of the cell lines, we found several metabolic proteins that changed upon ATX-101 treatment. Notably, most of these proteins responded to ATX-101 with opposite trends in TK16 versus TK12, i.e., the most sensitive and the least sensitive cell line (Figure 4C, heat map). We then performed the same analysis for additional pathways/sub-pathways including glycolysis, energy metabolism, proteasome and ubiquitin processes, PI3K/AKT/mTOR, MAPK and STAT signalling and apoptosis, and we detected that pathways with the most pronounced increase in TK12 had no/low increase or decrease in TK16 and the other sensitive cell lines (Figure 4D; complete lists of the pathway analysis are shown in Supplementary Figure S5). Of particular interest for metabolism is the increase in phosphofructokinase (PFKP), a rate-limiting enzyme in glycolysis, which is strongly increased in the least sensitive cell line TK12. Increased pull-down in TK12, but not in TK13, 14 and 16, was also detected for other proteins involved in energy metabolism, proteasome and several cellular signalling pathways (PI3K/AKT/mTOR, MAPK, STAT) as well as in several antiapoptotic proteins (Figure 4D).

In summary, these results show that targeting PCNA with ATX-101 affects multiple signalling pathways. The outcome is different in different cells, both in the same cell type, in cells from the same patient and in cells with similar sensitivity to ATX-101. No single protein or pathway response can fully explain the difference in sensitivity; however, the results indicate that ATX-101-resistant cells, have a stronger ability to activate primary metabolism and key signalling pathways than sensitive cells.

### 3.5. The Metabolic Shift in TK Cells After ATX-101 Treatment Correlates with Sensitivity

**Figure 5:**
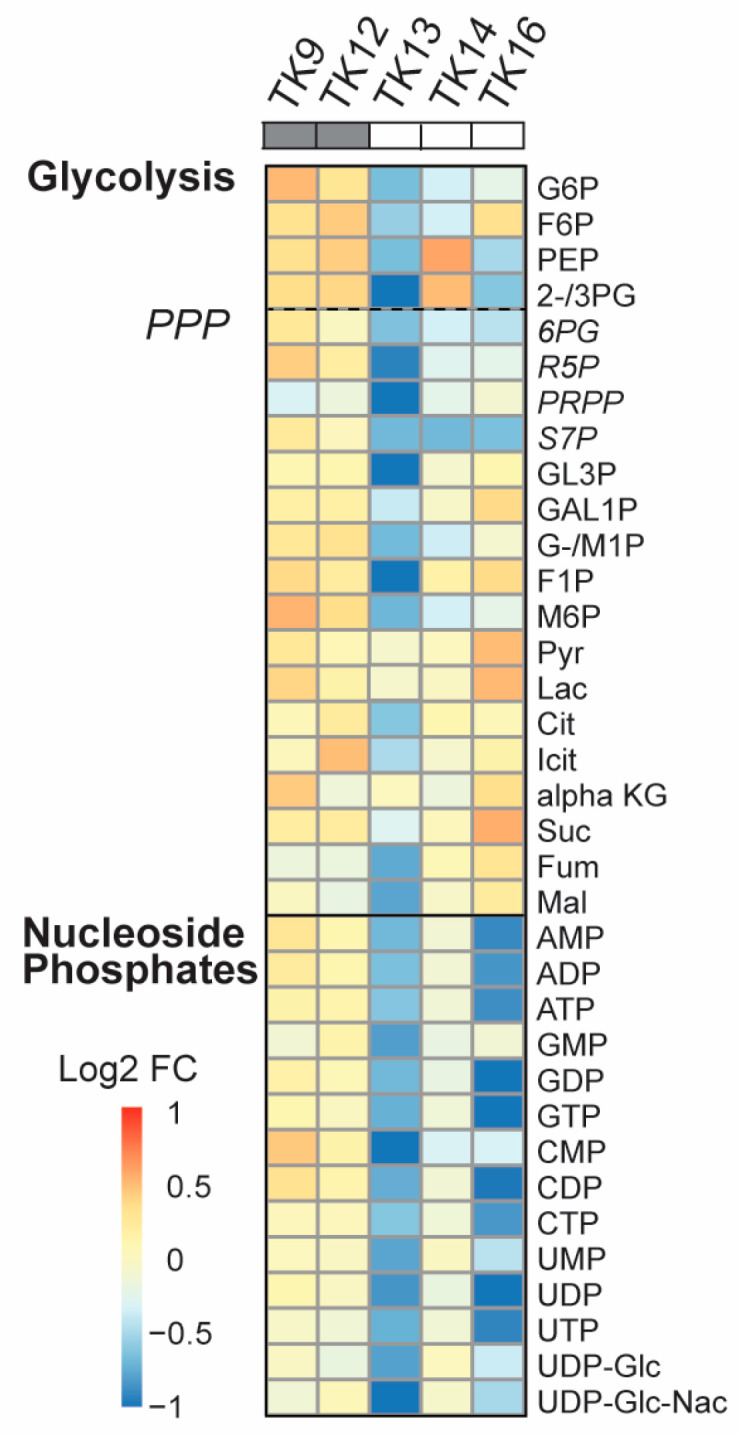
Reduced primary metabolism detected after ATX-101 treatment in ATX-101-sensitive cell lines. Log2 fold change (FC) of central carbon metabolites relative to untreated control measured in TK9, TK10, TK12, TK13, TK14 and TK16 cell lines treated with ATX-101 (10 µM) for 4 h. Average given relative to untreated control. Cell lines over grey boxes are less sensitive to ATX-101. Values are from *n* = 2–3 independent experiments. All sample concentrations are normalised to total protein in the extracts.

Data has indicated that the UPR is linked to metabolic reprogramming via activation of PDK1 and promotion of aerobic glycolysis [53]. Because we previously observe a “metabolic shift” with reduced metabolite pools in JJN3 and other haematological cell lines upon ATX-101 treatment [6], we next measured how ATX-101 treatment affected the metabolite pools in glycolysis, the PPP and the nucleoside phosphates in the TK cells. A metabolic shift was found in TK13-16 but not in TK9 and TK12 (Figure 5). That TK12 is less affected than TK13 and 14 indicates that the metabolic response to ATX-101 correlates with the sensitivity and not with the origin/genetic background of the cells. Previously, a metabolic shift following ATX-101 treatment was found in LPS-stimulated but not in unstimulated monocytes from the same donor [6]; thus, this suggests that the overall stress/activation level of the cells is important for ATX-101 activity. When ATX-101 was combined with the “stress inducer” melphalan, we detected a shift in the metabolite pools also in TK12 and TK18 cells; however, there was still not a shift in TK9 and TK10 cells (Supplementary Figure S4).

### 3.6. Sensitivity to ATX-101 Correlates with Reduced NAD+ and NADH Levels

**Figure 6:**
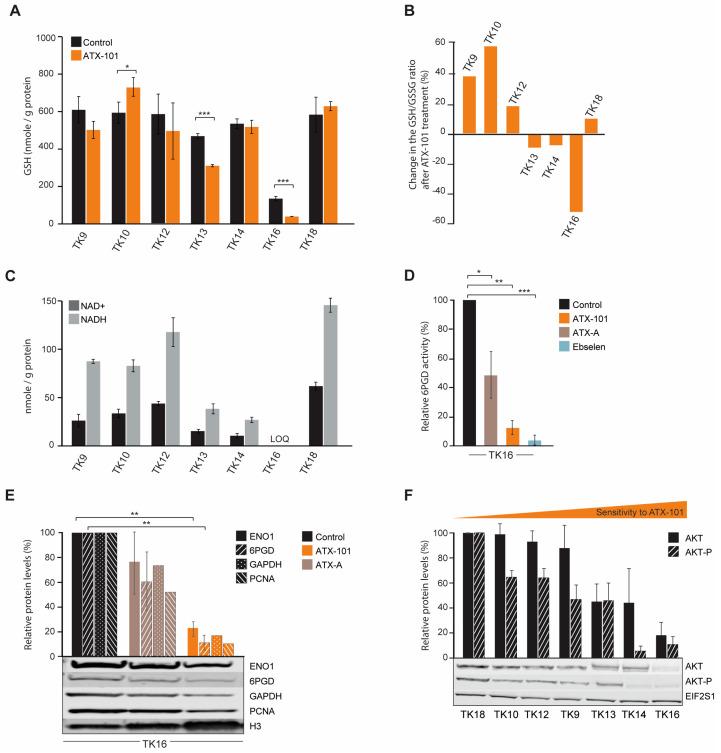
The GSH/GSSG ratio is reduced after ATX-101 treatment in the TK cell lines that also respond to treatment with a metabolic shift. (**A**) Intracellular GSH levels (nmol/g protein) in untreated and ATX-101-treated (10 µM, 4 h) TK cell lines. Mean ± SD from three replicate cultures. (**B**) Change relative to untreated control (%) in GSH/GSSH ratio in ATX-101 treated (10 µM, 4 h) TK cell lines. Mean from three replicate cultures. (**C**) Endogenous intracellular NAD+ and NADH levels (nmol/g protein) in TK cell lines. Mean ± SD from three replicate cultures. TK16 levels < limit of quantification (LOQ). (**D**) 6PGD activity in TK16 cells treated with ATX-101 (orange, 10 µM), ATX-A (brown, 10 µM) or the 6PGD-inhibitor ebselen (blue, 30 µM) for 4 h relative to activity in untreated control cells. Data displayed are mean ± SD (*n* = 3). (**E**) Protein levels of ENO1, 6PGD, GAPDH and PCNA in TK16 cells treated with ATX-101 (orange, 10 µM) or ATX-A (brown, 10 µM) for 24 h. Protein levels are normalised against H3 levels and relative to protein levels in untreated control cells. Data are displayed as mean ± SD (*n* = 3, ENO1 and 6PGD) or just mean (*n* = 2, GAPDH and PCNA). Representative Western blots are shown below the bars. (**F**) Protein levels of AKT and AKT-p in the panel of untreated TK cell lines arranged by increase in sensitivity towards ATX-101. Proteins are normalised to EIF2S1 levels and presented as relative to TK18 levels as mean ± SD (*n* = 3). * *p* < 0.05, ** *p* < 0.01, *** *p* < 0.001, unpaired two-tailed Student *t*-test. Representative Western blots are shown below the bars. Raw data and intensity measurements of Western blots in (**E**) and (**F**) are shown in Supplementary Figures S8 and S9, respectively.

Intracellular GSH levels were reduced after ATX-101 treatment in the MM cells JJN3 and RPMI 8226 (Figure 1C). In the TK cell lines, we did not find any clear correlation between the GSH response and sensitivity to ATX-101 (Figure 6A). Still, the GSH/GSSG ratio, which is the true measure of a cell’s redox capacity, was reduced after ATX-101 treatment only in TK13, 14 and 16, i.e., the three most sensitive cell lines (Figure 6B). The strongest reduction was observed in TK16, which also showed the strongest reduction in the nucleotide pools after ATX-101 treatment (Figure 5). The ability to reduce GSSG to GSH and to maintain the redox capacity of the cell depends on available NADPH, which is mainly produced in PPP. Since several metabolites in PPP are reduced in the sensitive cell lines after ATX-101 treatment, these effects may be related (Figure 5). No clear trends in intracellular NADP+/NADPH levels were found after ATX-101 treatment of the TK cell lines (Supplementary Figure S6); however, intracellular NAD+ and NADH levels in untreated cells were lowest in the three cell lines with the most pronounced metabolic shift and highest sensitivity (Figure 6C). Low endogenous levels of NAD+ were also detected in the two MM cell lines JJN3 and RPMI 8226; therefore, low NAD+ and NADH levels may predict sensitivity to ATX-101.

Low levels of NAD+ and NADH can be related to reduced glycolysis and/or reduced PRPP pools (Figure 5), as PRPP is required for new synthesis of NAD+. We recently showed that both the protein level and the specific enzyme activity of 6PGD, the third enzyme in PPP, is reduced by ATX-101 treatment in JJN3 cells [6]. Here, we show a strong reduction in 6PGD activity also in TK16 cells after ATX-101 treatment (Figure 6D).

Like in other haematological cells studied [6], ATX-101 treatment of TK16 cells reduced the protein levels of ENO1 and GAPDH (Figure 6E). GAPDH has recently been shown to be of particular interest for the oxidative PPP; therefore, reduced GAPDH not only reduces the glycolysis but also likely directly reduces the redox capacity of the cell [54]. In HAP1 and JJN3 cells, a small reduction in PCNA was detected after ATX-101 treatment [6]; however, similar to glioblastoma cells [55], a strong reduction in PCNA levels was detected in TK16 cells upon ATX-101 treatment (Figure 6E).

Next, we investigated whether baseline protein levels of AKT and phosphorylated-AKT (AKT-P) in the TK cell lines could predict sensitivity to ATX-101. We found that the most sensitive cells (TK13, 14 and 16) had the lowest levels of AKT and the lowest levels of AKT-P (Figure 6F); thus, there might be a correlation. However, no uniform response of AKT-P after ATX-101 treatment was detected, and this differs from the clear reduction of AKT-P detected after ATX-101 treatment in four glioblastoma multiforme cell lines [55]. We detected no correlation between ATX-101 sensitivity and PCNA or ENO1 protein levels, but the protein level was lowest in the most sensitive cell line, TK16 (Supplementary Figures S7 and S10).

A clear degradation of both 6PGD and ENO1 was observed after ATX-101 treatment of TK16 (Figure 6E). Reduction of these proteins may have implications for the immunological status of, for example, the tumour microenvironment (TME). Recent data suggested that ENO1 and 6PGD are interesting targets in combination with both immunotherapy and chemotherapy, i.e., inhibition of both enzymes was shown to induce metabolic reprogramming beneficial for therapeutic efficacy [20,22,56]. 6PGD inhibition was shown to enhance CD8+ T-cell antitumour function [56], and ENO1 inhibition was shown to increase bone marrow plasmacytoid dendritic cell-induced MM-specific CD8+ CTL and NK activity in co-cultures [20]. The latter was further enhanced in combination with immune checkpoint therapy. ENO1 expression was shown to be increased in both monoclonal gammopathy of undetermined significance (MGUS) and in active MM [21], and the ENO1 expression level was also shown to correlate with the outcome in several different cancers, including MM [20,57,58]. Bone marrow plasmacytoid dendritic cells–MM interactions stimulate ENO1 expression and elevated levels of ENO1 are found in immunosuppressive myeloid-derived suppressor cells [20]. ATX-101 has shown good antitumour efficacy in co-culture of primary patient MM cells and bone marrow stromal cells (BMSC) [13]; however, ATX-101 inhibits multiple PCNA–protein interactions, not only ENO1–PCNA and 6PGD–PCNA, and it is therefore difficult to predict exactly how ATX-101 will affect the MM real TME in the bone marrow. However, the prolonged disease stabilisation seen in 70% of the efficacy population in the recent Phase I trial in advanced solid cancers (different cancers) may suggest that ATX-101 has immunomodulating activity [18].

### 3.7. Biomarkers for ATX-101-Sensitive MM Cells

**Figure 7:**
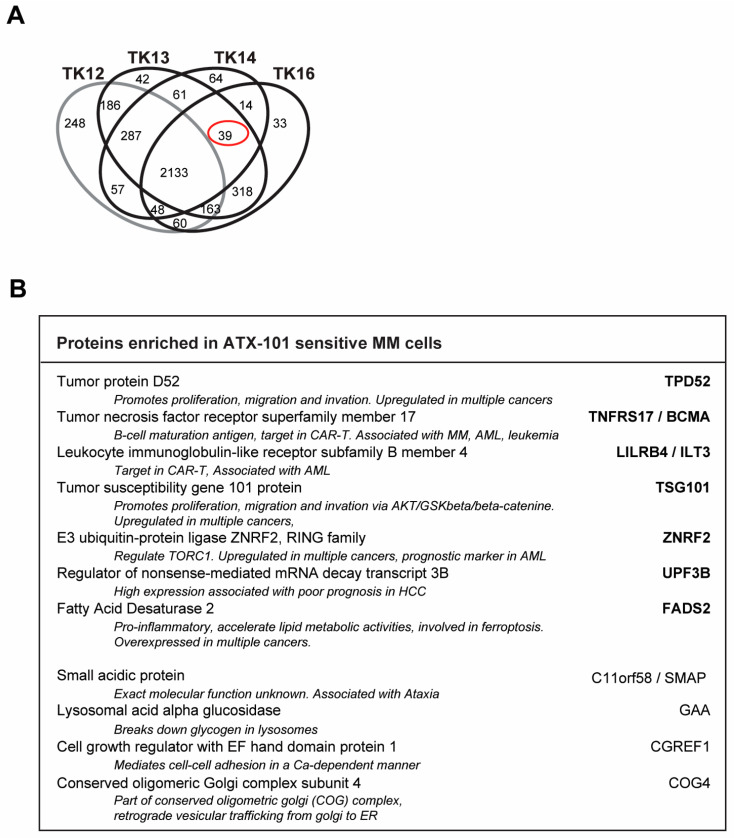
Potential biomarkers for ATX-101-sensitive MM cells. (**A**) Venn diagram of proteins pulled down from untreated TK12, TK13, TK14 and TK16 cell extracts using the MIB assay. Only proteins that are significantly changed from untreated control (similar change in extracts from all three repeated experiments, Wilcoxon sign rank test) are shown. Full data set is deposit in PXD033510. (**B**) Proteins out of the 39 proteins marked with red ring in A which are also detected in JJN3 but not in MCCAR, NB4, HL60, primary human monocytes from three donors [6], PXD028314, PXD017474), UmUc-3, T24 ([5] PXD011044), U2OS, H460, A549 [30] (PXD005286) or TK9 (PXD033531). Only proteins that are significantly changed from untreated control (similar change in extracts from all three repeated experiments, Wilcoxon sign rank test) are included.

Analysis of the signallome in JJN3 cells and in the sensitive TK cell lines showed that several pathways including ER and proteasomal processing, metabolism, translation regulation and oxidative phosphorylation are affected by ATX-101 treatment. We therefore next investigated which proteins were pulled down from untreated TK13, TK14 and TK16 cell extracts but not from the less sensitive TK12 cell line (including only significant proteins, i.e., proteins pulled down in all three independent biological replicates). These proteins could represent proteins that are endogenously activated only in sensitive cells. We found 39 proteins (Figure 7A, red circle, full list of proteins in Supplementary Table S2), and STRING analysis of these proteins showed that they belong to different biological processes but were not enriched in specific pathways. Of interest for the regulation of primary metabolism was the increased pull-down of Mucin-1, which has been shown to increase the expression of several glycolytic genes, including ENO1, in a HIF-1 dependent manner [59] (Supplementary Table S2).

We also checked to see if any of these proteins were found in pull-downs from other cancer cell lines tested in our laboratory using the MIB assay. The cell lines examined were as follows: (i) the MM cell line JJN3, the B-lymphoblastoid cell line MCCAR, the acute myeloid leukaemia cell lines NB4 and HL60 and primary human monocytes from three donors [6], (ii) the bladder cancer cell lines UmUc-3 and T24 [5], (iii) the sarcoma cell line U2OS and the two lung cancer cell lines H460 (non-small cell lung cancer, NSCLC) and A549 (squamous cell lung cancer) [30] and (iv) the TK9 cell line (this study). We found that 11 out of the 39 proteins were also pulled down from JJN3 but not from any of the other cell lines (Figure 7B). This may indicate that activation of these proteins correlates with ATX-101 sensitivity specifically in MM cells.

Seven of these proteins are dysregulated in several different cancers, and some are known targets for cancer therapy, including in MM. These proteins are as follows: (i) tumour protein D52 (TPD52), which regulates AMPK activity [60] and whose overexpression is associated with poor prognosis in multiple cancers [61]; (ii) tumour necrosis factor receptor superfamily member 17/B-cell maturation antigen (TNFRS17/BCMA), a known target for several cancer therapies, including CAR-T in relapsed MM patients [62,63]; (iii) leukocyte immunoglobulin-like receptor-B4 (LILRB4/ILT3), a potential target for immune checkpoint therapy as it is important for myelosuppression via interaction with fibronectin [64,65]; (iv) tumour susceptibility gene 101 (TSG101), part of the ESCRT-1 endosomal sorting [66,67] and shown to be important in regulating the AKT/GSK3beta/beta-catenin and RhoC/Cofilin pathways [68] and recently identified as critical for PARP1-activation [69]; (v) RING E3 ubiquitin ligase ZNRF2, which is regulated by the AKT/mTOR pathway and is frequently upregulated in cancer [70] and has been suggested as a prognostic marker for cervical squamous cell carcinoma, AML and NSCLC [70,71,72]; (vi) up-frameshift suppressor 3 (UPF3B), which is a regulator of nonsense-mediated mRNA decay and is part of a set of DNA repair-related five-gene signature in oesophageal cancer, which is upregulated in several other cancers [73,74,75] and is identified as an immunotherapeutic target and a prognostic biomarker in several tumours [75]; and (vii) fatty acid desaturase 2 (FADS2), the first rate-limiting enzyme in the biosynthesis of polyunsaturated fatty acids; FADS2 is aberrantly expressed in several cancers, promotes proliferation, inhibits ferroptosis and induces inflammation in the TME [76]. All these proteins are important in regulating both cancer growth and cancer–TME interactions. Less is known about the role of the last four proteins, but recent reports have identified small acidic protein (C11orf58/SMAP) as part of a 12-gene m6A-related risk signature in melanoma [77] and cell growth regulator with EF hand domain protein 1 (CGREF1) as a potential biomarker in prostate, osteosarcoma and cervical cancers [78,79,80]. The lysosomal acid alpha glucosidase GAA is not known to be associated with cancer, but conserved oligomeric Golgi complex subunit 4 (COG4), which is involved in vesicular trafficking from the Golgi to the ER, is upregulated in renal clear cell carcinoma [81].

Interestingly, TPD52 [82], TNFRS17/BCMA [83], TSG101 [84], FADS2 [85] and GAA [86], i.e., 5 out of 11 proteins, have been previously implicated in ER stress and/or redox balance. None of these 11 proteins were found among the genes that were overexpressed in the sensitive compared to the non-sensitive cells. The fact that these proteins were found in the pull-down in the MIB assay but were not overexpressed suggests that they are activated. Altogether, these 11 proteins could potentially present biomarkers for MM cells with high ER stress and thereby high sensitivity to ATX-101 treatment.

### 3.8. No Cross-Resistance Between the Protease Inhibitor Carfilzomib and ATX-101

**Figure 8:**
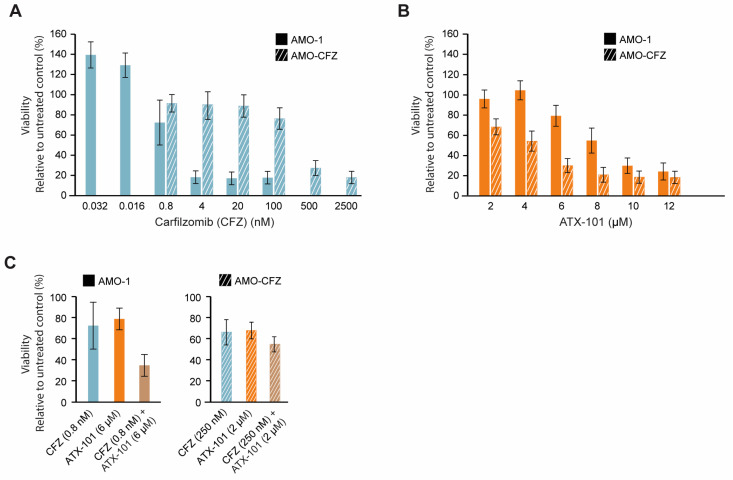
Proteasome inhibitor-resistant MM cells remain sensitive to ATX-101. Dose response of carfilzomib (0.032 to 2500 nM, blue) (**A**) and ATX-101 (2–12 μM, orange), (**B**) in sensitive (AMO-1, blank) and carfilzomib (CFZ)-resistant (AMO-CFZ, diagonal stripes) AMO-1 cells. (**C**) Single agent and combination treatments of CFZ and ATX-101; 0.8 nM CFZ and 6 μM ATX-101 on AMO-1 ((**left**) panel) and 250 nM CFZ and 2 μM ATX-101 on AMO-CFZ cells ((**right**) panel). All data presented are from the PrestoBlue assay on day 4 after treatment. The average of 4–6 replicate wells from one representative out of two repeated experiments is shown. Data are normalised to untreated control.

Increased ER stress is caused by the UPR and reduced flux through the ER, and therefore, high antibody production makes MM cells particularly vulnerable to this type of stress. Further inhibition of the proteasome system, for example, by proteasome inhibitors or the secretory/vehicle system, will further increase this stress and may therefore eventually lead to apoptosis. Proteasome inhibitors are therefore often good anticancer drugs for MM patients, and they gave early on hope for a curative treatment; however, resistance eventually occurs in most patients [24]. Any drug that can target ER stress in a different way and/or re-sensitise MM cells could therefore be of clinical interest; therefore, we next investigated whether ATX-101 has any cross-resistance with carfilzomib (CFZ), a proteasome inhibitor in clinical use. Dose-response experiments in two AMO-1 MM cell lines, one resistant (AMO-CFZ) and one sensitive (AMO-1) [26] (Figure 8A), showed that ATX-101 treatment was at least as efficient in AMO-CFZ as in AMO-1 (Figure 8B). The resistance mechanism of AMO-CFZ is not directly caused by mutations in the proteasome (no PSMB5 gene mutations) but by the IRE-1/XBP1 “low pattern” of UPR activations often found in cells from resistant patients and in proteasome inhibitor-resistant MM cell lines [26]. Extensive proteomic analysis has shown that resistance in the AMO-CTZ cells is associated with changes in the expression levels of hundreds of proteins, including proteins involved in energy metabolism, protein folding or degradation, and, interestingly, similar changes have been found in AMO cells resistant to bortezomib, another proteasome inhibitor [26]. The ATX-101 sensitivity of AMO-CFZ cells supports that the two drugs have different modes of action. This is further supported by the additive effects observed when ATX-101 and CFZ were combined (Figure 8C).

One reason for the trend towards an increased efficacy of ATX-101 in the CFZ-resistant cells may be that these cells have increased overall stress, probably including ER stress, and that ATX-101 impairs other and/or counteracts the mechanism that these cells use to escape the proteasome inhibition. For example, ATX-101 treatment has been shown to impair vesicle trafficking and alter the regulation of apoptosis, signalling and primary metabolism (here and [3,5,6,7,13,14]), all mechanisms that have been implicated in the development of resistance to proteasome inhibitors (reviewed in [87]). These data support further exploration of the use of ATX-101 in combination with proteasome inhibitors in relapsed patients with a prior good response to proteasome inhibitors.

## 4. Conclusions

In this comprehensive multi-omics study of the effect of targeting PCNA with the experimental drug ATX-101 in 10 MM cell lines, we find that high ribosomal gene expression, proteasome and ER stress and reduced redox capacity are indicators of high sensitivity to ATX-101. In addition, treatment with ATX-101 further increases ER stress and reduces glycolysis and PPP. This suggests a central role of PCNA in the regulation of these stress responses. Eleven proteins that were pulled down only from sensitive MM cells may be indicators of this responsive stress status in MM cells.

## Figures and Tables

**Table 1:** TK cell line characteristics. Cell line, site of isolation and culture characteristics of TK cell lines. Cells were isolated from peripheral blood (PB), bone marrow (BM) or a pleural effusion in the pleural cavity of the lungs (PE) of MM patients.

Cell Line	Cytogenetics	Treatment	Site of Isolation	Culture Characteristics
TK9	t(4:14)	A, PI, iMIDs, ASCT, T	PB	Semi-adherent
TK10	t(4;14), t14;16), 13q-, 17p-, 1q+	A, PI, CD38, ASCT, T	PB	Semi-adherent
TK12 *	Hypotetraploidity, chr 13-, t(14;16), 17p-	A, PI, ASCT	PE	Semi-adherent
TK13 *	Same as 12	+ iMIDS	PE	Semi-adherent
TK14 *	Same as 12		PE	Suspension
TK16	No information	A, PI, iMIDs, T	BM	Suspension
TK18	Normal karyotype	A, PI, iMIDs, ASCT	BM	Semi-adherent

* Isolated from the same donor. A higher number indicates increasing time passed since diagnosis. Cytogenetics, lack of chromosomes/chromosome fragments means monoallelic loss. Alkylator (A), proteasome inhibitors (PI), immunomodulatory agents (iMIDs), autologous stem cell transplant (ASCT), topoisomarase I (T).

## Data Availability

The proteomic data for the TK cell lines are deposited in PRIDE, project IDs PXD033510 (https://www.ebi.ac.uk/pride/archive/projects/PXD033510/, TK12, 13, 14 and 16) and PDX033531 ((https://www.ebi.ac.uk/pride/archive/projects/PXD033531, TK9), both accessed on 28 April 2022. Raw data from the MM cell line JJN3, the B lymphoblastoid cell line MCCAR, the acute myeloid leukaemia cell lines NB4 and HL60 and primary human monocytes from three donors (also described in [6]) have project IDs PXD028314, PXD017474; from the bladder cancer cell lines UmUc-3 and T24 (also described in [5]), PXD011044; the sarcoma cell line U2OS and the two lung cancer cell lines H460 (non-small cell lung cancer, NSCLC) and A549 (also described in [30]) PXD005286, and the RNA sequences at The Sequence Read Archive (BioProject ID PRJNA1176350, http://www.ncbi.nlm.nih.gov/bioproject/1176350, accessed on 23 October 2024).

## Supplementary Materials

The following supporting information can be downloaded at: https://www.mdpi.com/article/10.3390/cancers16233963/s1, Supplementary Figure S1: Treatment with ATX-101 affects proteins regulating multiple signalling pathways; Supplementary Figure S2: Targeting PCNA with ATX-101 alters metabolite pools, metabolic enzymes and associated signalling pathways in JJN-3 cells; Supplementary Figure S3: Elevated levels of proteins involved in ER stress in ATX-101-sensitive cell lines; Supplementary Figure S4: Intracellular levels of nucleoside phosphates, and glycolytic and PPP intermediates decrease more in melphalan–ATX-101 combination-treated TK cell lines than in TK cells treated with only ATX-101; Supplementary Figure S5: ATX-101 treatment affects multiple signalling pathways in all cell lines tested; Supplementary Figure S6: Endogenous NADP+ and NADPH levels are similar between TK cell lines; Supplementary Figure S7: No correlation between PCNA and ENO1 levels and sensitivity to ATX-101; Supplementary Figure S8: Western blots and intensity of bands related to Figure 6E; Supplementary Figure S9: Western blots and intensity of bands related to Figure 6F; Supplementary Figure S10: Western blots and intensity of bands related Supplementary Figure S7; Supplementary Table S1: IC50 of the cell lines used in this paper; Supplementary Table S2: Proteins pulled down from ATX-101-sensitive TK cell lines.

## Abbreviations

6PG, 6-phosphogluconate; 6PGD, 6-phosphogluconate dehydrogenase; APIM, AlkB homologue 2 PCNA-interacting motif; BM, bone marrow; capIC, capillary ion chromatography; DE, differentially expressed; EC, adenylate energy charge; ENO1, enolase 1; ER, endoplasmic reticulum; ERAD, ER-associated degradation G6P, glucose 6-phosphate; G6PDH, glucose-6-phosphate dehydrogenase; ID, isotope dilution; LC, liquid chromatography; LPS, lipopolysaccharide; MIB, multiplexed inhibitor beads; MM, multiple myeloma; MS, mass spectrometry; MS/MS, tandem mass spectrometry; PB, peripheral blood; PCNA, proliferating cell nuclear antigen; PE, pleural effusion; PIP-box, PCNA-interacting protein-box; PPP, pentose phosphate pathway; TLR, Toll-like receptor; PTM, post-translational modification; TCA, tricarboxylic acid; UPR, unfolded protein response.
