# Supplementary material for: Multiple Myeloma Cells with Increased Proteasomal and ER Stress Are Hypersensitive to ATX-101, an Experimental Peptide Drug Targeting PCNA
Source: Cancers (Basel). 2024 Nov 26;16(23):3963. doi: 10.3390/cancers16233963 (PMC11640687; doi:10.3390/cancers16233963)
Supplement: Supplementary file 1 [file cancers-16-03963-s001.zip › cancers-3312807-supplementary.pdf]

Supplementary Materials

Multiple Myeloma Cells with Increased Proteasomal and ER Stress Are Hypersensitive to ATX-101, an Experimental Peptide Drug Targeting PCNA

Camilla Olaisen [1,†], Lisa Marie Røst [2,†], Animesh Sharma [3], Caroline Krogh Søgaard [1], Tiffany Khong [4,5], Sigrid Berg [1], Mi Jang [2], Aina Nedal [1], Andrew Spencer [4,5], Per Bruheim [2] and Marit Otterlei [1,6,7,*]

[1] Department of Clinical and Molecular Medicine, Faculty of Medicine and Health Sciences, NTNU Norwegian University of Science and Technology, NO-7491 Trondheim, Norway; camilla.olaisen@stolav.no (C.O.); caroline.d.sogaard@ntnu.no (C.K.S.); sigrbe@ntnu.no (S.B.); aina.nedal@ntnu.no (A.N.)

[2] Department of Biotechnology and Food Science, Faculty of Natural Sciences, NTNU Norwegian University of Science and Technology, NO-7491 Trondheim, Norway; lisa.marie.rost@gmail.com (L.M.R.); kia3111@gmail.com (M.J.); per.bruheim@ntnu.no (P.B.)

[3] Proteomics and Modomics Experimental Core Facility (PROMEC), NTNU Norwegian University of Science and Technology, NO-7491 Trondheim, Norway; animesh.sharma@ntnu.no

[4] Australian Centre for Blood Diseases, Monash University, Melbourne 3004, Australia; tiffany.khong@monash.edu (T.K.); andrew.spencer@monash.edu (A.S.)

[5] Department of Malignant Haematology and Stem Cell Transplantation, Alfred Hospital, Melbourne 3004, Australia

[6] Clinic of Surgery, St. Olavs Hospital, Trondheim University Hospital, NO-7006 Trondheim, Norway

[7] APIM Therapeutics A/S, Rådhusveien 12, NO-7100 Rissa, Norway

* Correspondence: marit.otterlei@ntnu.no

† These authors contributed equally to this work.

Supplementary Figure S1

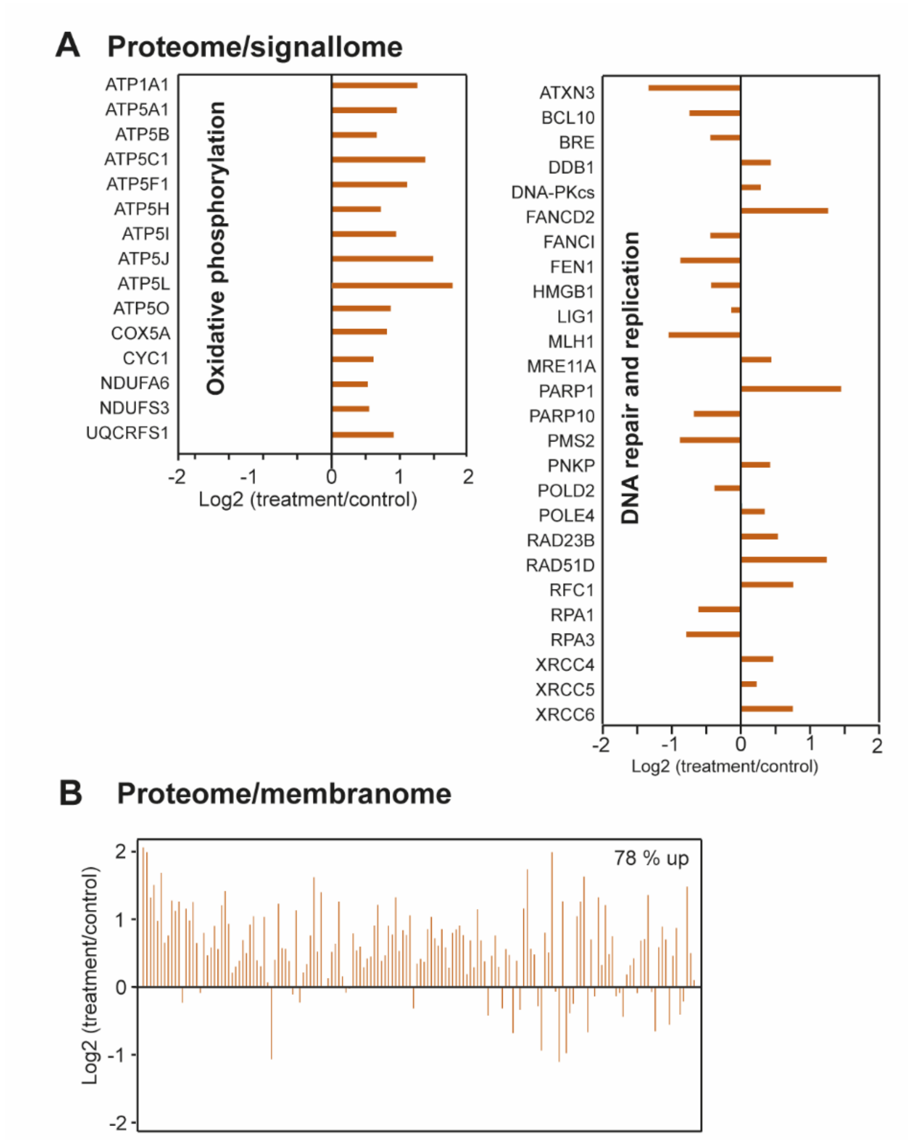

Supplementary Figure S1: Treatment with ATX-101 affects proteins regulating multiple signalling pathways (**A**) Results of MIB-assay of extract from JJN3 cells treated with ATX-101 (6 µM) for 4h. Left panel: proteins involved in ER/antioxidants/oxidative phosphorylation, right panel: protein involved in DNA repair and replication. Data shown is mean from three repeated experiments, presented as $\log_2$ fold change relative to untreated control. Significant according to the Wilcoxon Sign Rank test. Bolded proteins are mentioned in the text. (**B**) Transmembrane proteins detected by the MIB-assay. Proteins significantly changed from untreated control in at least one treatment according to the Wilcoxon Sign Rank test are shown. 78% of all deregulated transmembrane proteins detected by the MIB-assay are upregulated relative to control.

Supplementary Figure S2

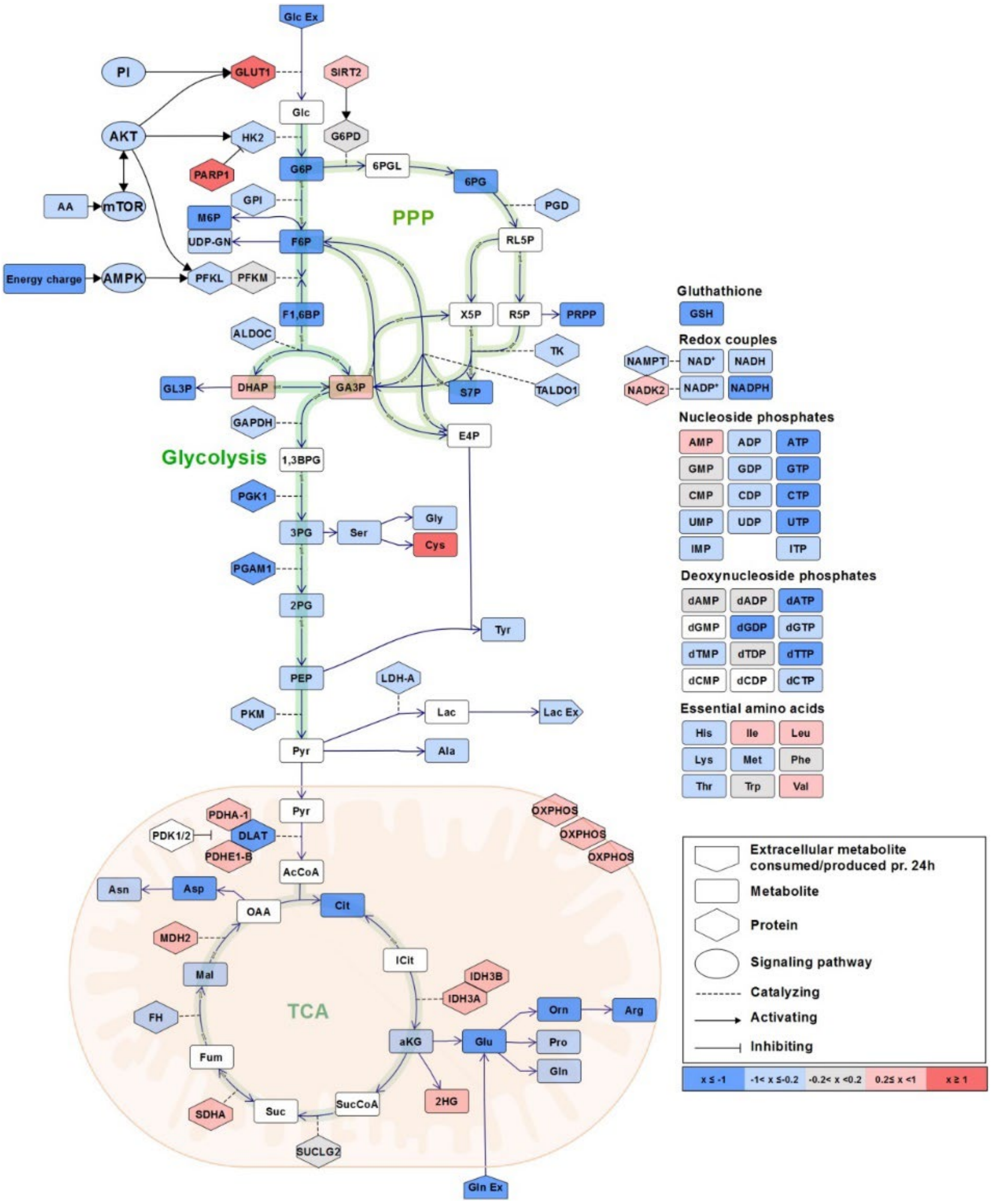

Supplementary Figure S2. Targeting PCNA with ATX-101 alters metabolite pools, metabolic enzymes and associated signalling pathways in JJN-3 cells. Log2 fold change of glycolytic, TCA and PPP intermediates, phosphorylated sugars, amino acids and (deoxy)nucleoside phosphates with associated proteins and pathways in JJN3 cells treated with ATX-101-peptide (8 µM) for 4 hours, normalized to cell density, given relative to untreated control. White colour indicates that metabolite/protein was not detected. For grouped metabolites and pathways, the average log2 is presented. The figure is based on data presented in Figure 1.

Supplementary Figure S3

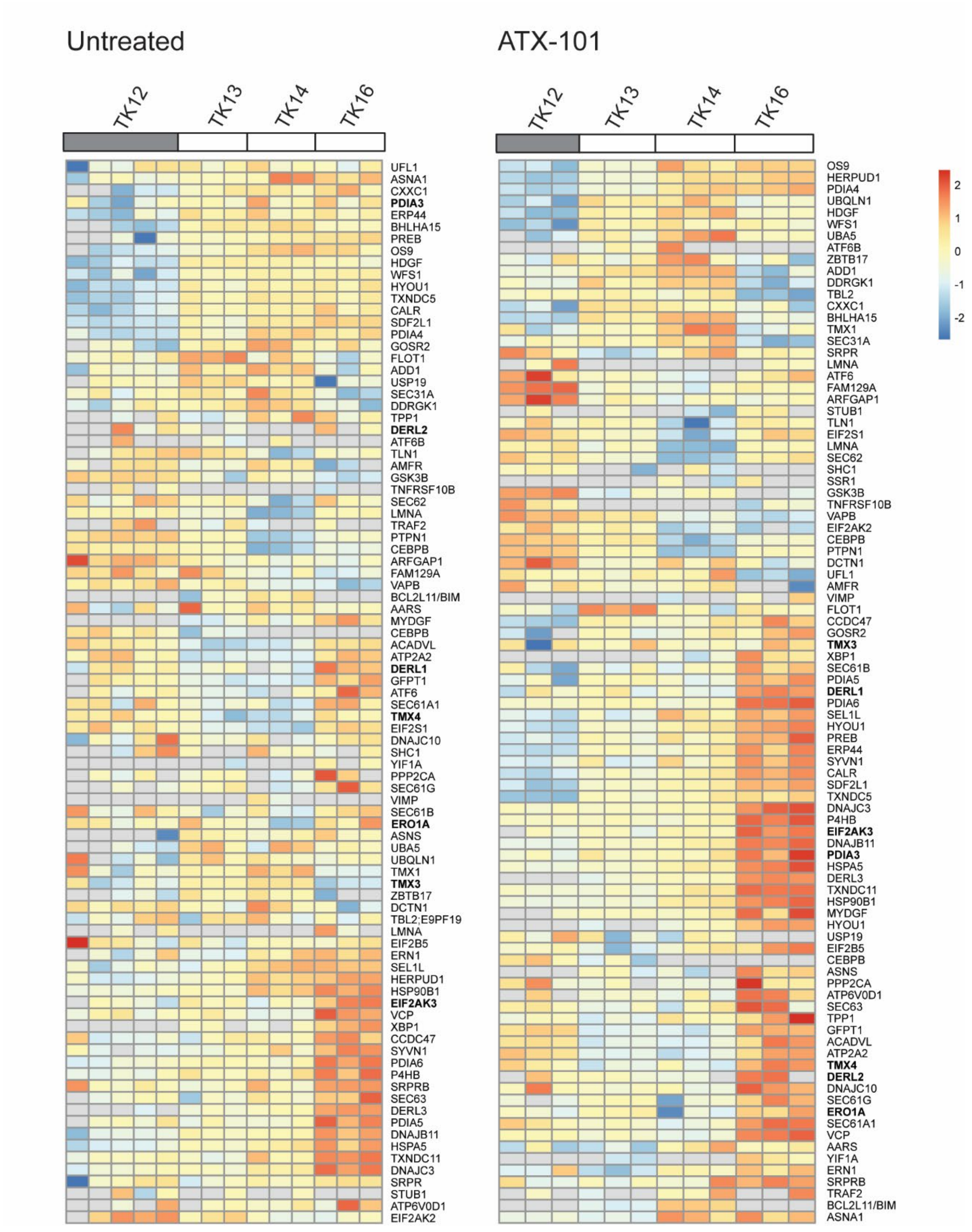

Supplementary Figure S3: Elevated levels of proteins involved in ER stress in ATX-101 sensitive cell lines. Cluster analysis of all proteins belonging to GO:003476, Response to ER stress, pulled down form untreated and ATX-101 treated cells. Cell line over grey box is less sensitive to ATX-101.Data from 3-5 independent biological replica are shown.

Supplementary Figure S4

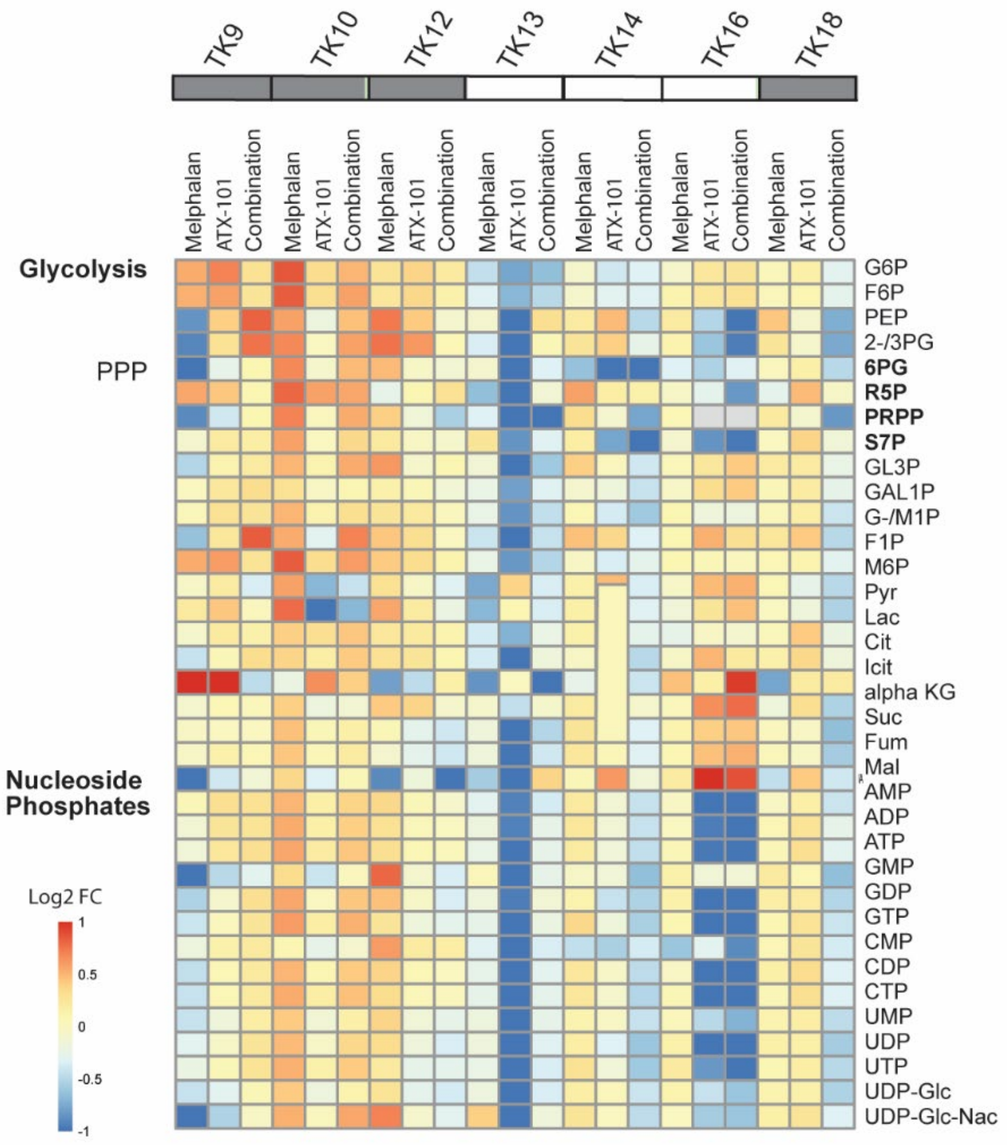

Supplementary Figure S4: Intracellular levels of nucleoside phosphates, and glycolytic and PPP intermediates decrease more in melphalan-ATX-101 combination treated TK cell lines than in TK cells treated with only ATX-101. Log_2 fold change in intracellular levels of central carbon metabolites measured in TK9, TK10, TK12, TK13, TK14, TK16 and TK18 cell lines treated with ATX-101 (10 µM), melphalan (1 µM) or the respective combination for 4 h. Cell lines over grey boxes are less sensitive to ATX-101. Sample concentrations are normalized to total protein in the same extract. Average from three replicate cultures are given relative to untreated control.

Supplementary Figure S5

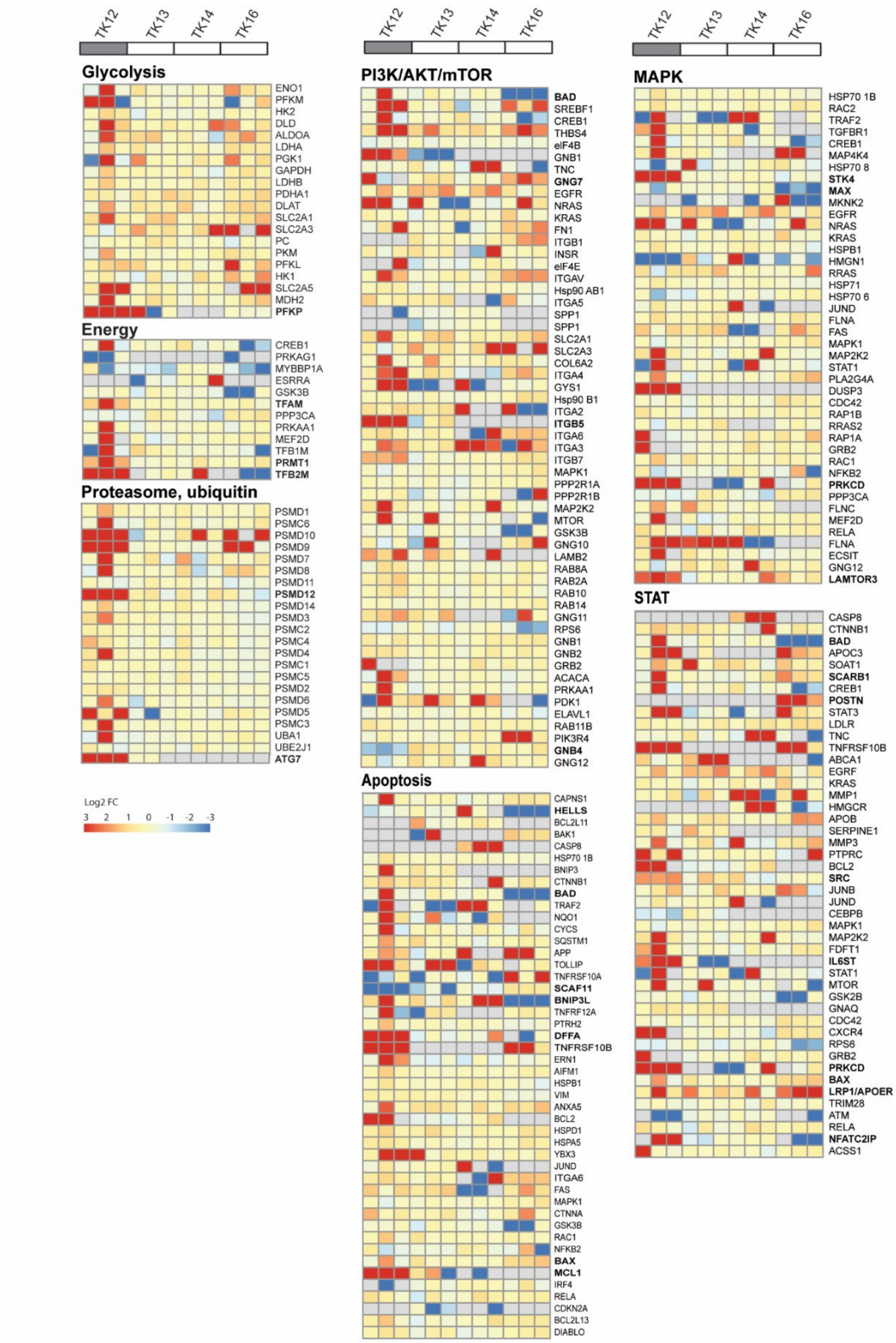

Supplementary Figure S5: ATX-101 treatment affects multiple signaling pathways in all cell lines tested. Heat map of proteins involved in glycolysis, energy metabolism, proteasome, PI3K/AKT/mTor, MAPK, STAT and apoptosis ATX-101 treated TK12, 13, 14 and 16. Proteins significantly changed in more than 1 of the sensitive cell lines are included. Cell line over grey box is less sensitive to ATX-101.

Supplementary Figure S6

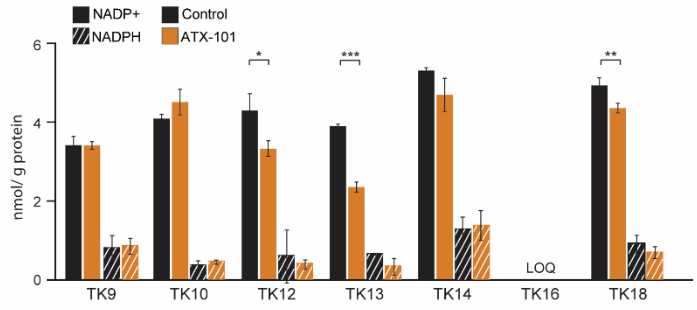

Supplementary Figure S6: Endogenous NADP+ and NADPH levels are similar between TK cell lines. Endogenous intracellular NADP+ and NADPH levels (nmol/g protein) in TK cell lines. Mean ± SD from three replicate cultures. TK16 levels < LOQ.

Supplementary Figure S7

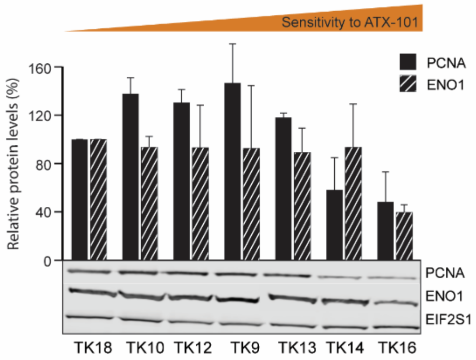

Supplementary Figure S7: No correlation between PCNA and ENO1 levels and sensitivity to ATX-101. Protein levels of PCNA and ANO1 in the panel of untreated TK cell lines arranged by increase in sensitivity towards ATX-101. Proteins are normalized to EIF2S1 levels and presented as relative to TK18 levels asmean ± SD (n=3). Representative western blots are shown below the bars. Raw data and intensity measurements of Western blots are shown in Supplementary Figure S10.

Supplementary Figure S8

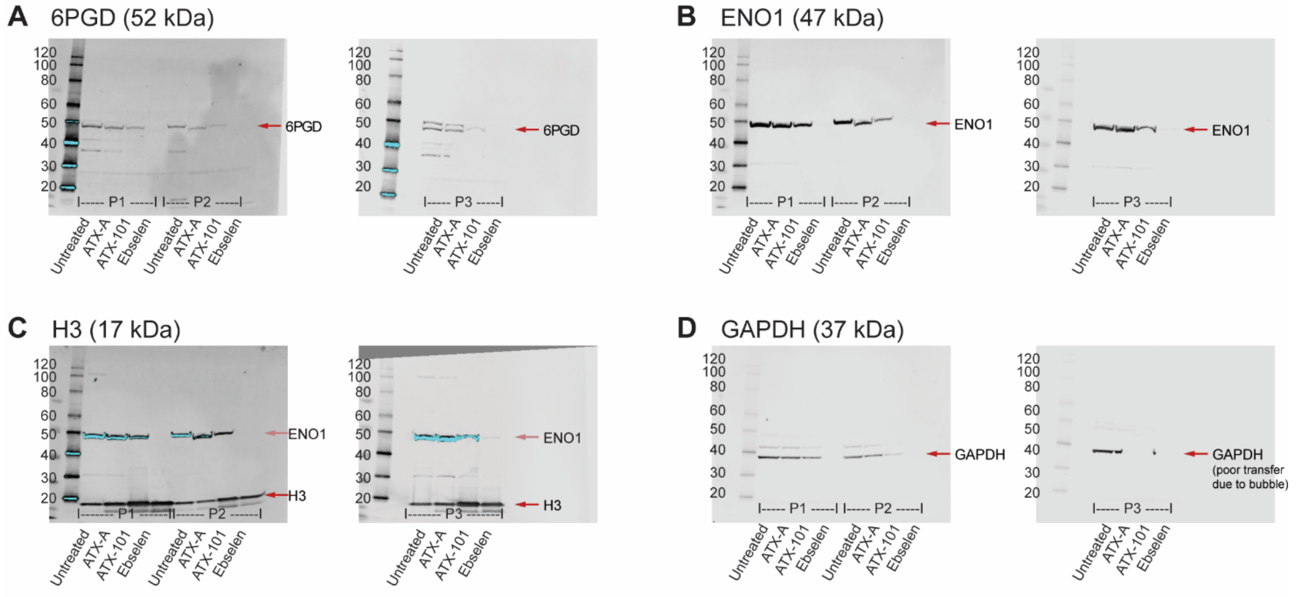

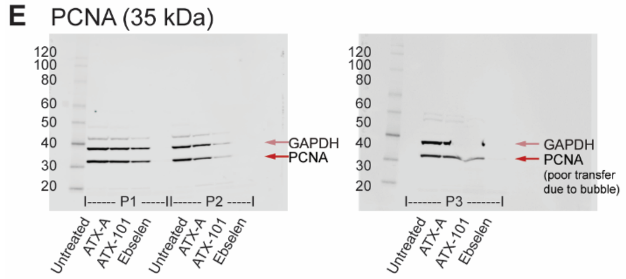

F Intensity of bands

TK16 cells:		Untreated	ATX-A	ATX-101
**ENO1**	P1	9 911 024	7 547 998	4 510 752
	P2	6 354 940	3 556 996	1 584 080
	P3	8 849 895	10 248 412	4 346 000
**6PGD**	P1	3 062 744	1 738 592	599 696
	P2	1 487 064	961 616	383 060
	P3	5 986 184	3 766 280	565 068
**GAPDH**	P1	1 291 112	852 421	514 927
	P2	677 387	515 790	105 041
	P3			
**PCNA**	P1	3 687 897	2 614 866	1 030 935
	P2	2 464 427	1 026 026	140 937
	P3	1 410 940	1 040 127	531 998
**H3**	P1	5 405 540	7 799 404	9 199 648
	P2	4 024 756	3 036 056	6 212 552
	P3	4 037 611	4 535 259	7 705 944

Supplementary Figure S8: Western blots and intensity of bands related to Figure 6E.

Supplementary Figure S9

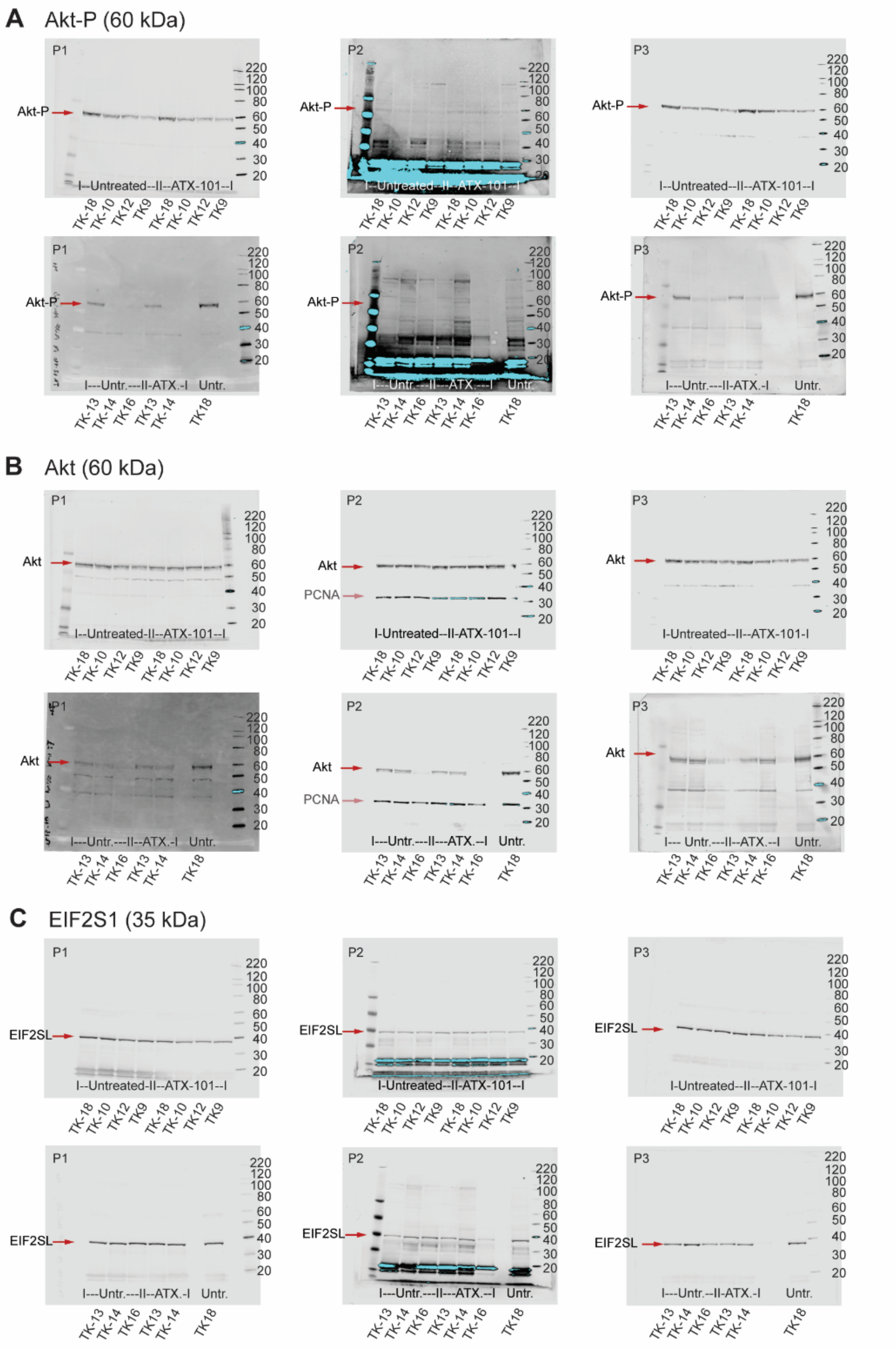

D Intensity of bands

Untreated cells:		Membrane 1				Membrane 2			
		TK18	TK10	TK12	TK9	TK13	TK14	TK16	TK[illegible]
Akt-P	P1	2 851 776	1 591 171	1 183 001	703 414	1 663 504	106 592	185 568	5 034
	P2	510 408	305 232	263 256	257 616	117 456	17 648	31 244	270
	P3	1 481 229	847 950	925 873	704 082	618 843	68 373	78 766	1 376
Akt	P1	1 441 221	1 373 581	1 038 241	920 117	629 696	382 768	127 488	1 941
	P2	5 336 415	5 241 841	4 147 925	5 354 407	2 281 344	2 336 348	906 149	4 861
	P3	2 236 933	1 638 896	1 634 842	1 404 301	1 230 720	1 217 734	281 312	2 773
EIF2SL	P1	3 037 536	2 848 414	2 242 734	2 247 032	3 103 092	3 172 973	3 388 775	3 367
	P2	1 797 420	1 671 103	1 420 188	1 688 400	705 314	1 191 184	1 319 538	1 837
	P3	1 748 696	1 432 263	1 542 417	1 560 834	1 421 643	2 063 832	810 511	1 766

Supplementary Figure S9: Western blots and intensity of bands related to Figure 6F

Supplementary Figure S10

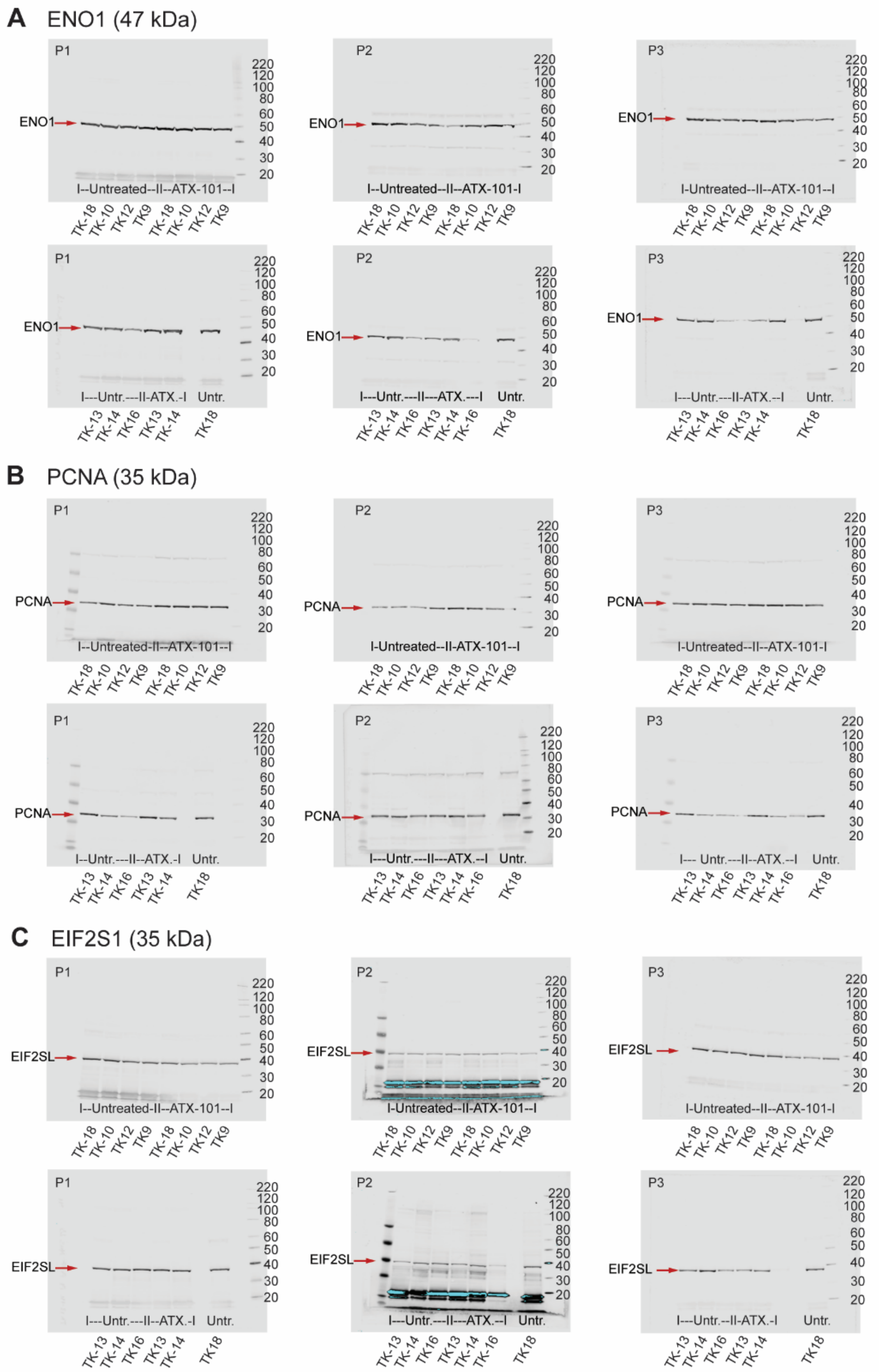

D Intensity of bands

Untreated cells:		Membrane 1 TK18	TK10	TK12	TK9	Membrane 2 TK13	TK14	TK16	TK18
ENO1	P1	4 263 128	4 141 737	4 182 233	4 654 307	3 083 129	2 960 900	1 585 326	4 455 782
	P2	4 753 044	3 975 341	2 455 722	2 010 548	2 297 189	2 563 686	769 063	2 934 722
	P3	1 625 113	1 160 233	1 159 072	1 237 918	1 284 620	1 355 029	331 667	1 542 525
PCNA	P1	3 144 527	4 326 789	3 109 118	3 108 217	3 211 977	1 553 170	868 368	2 857 608
	P2	936 762	1 068 000	874 670	1 615 009	2 297 189	2 563 686	769 063	1 758 334
	P3	2 996 171	3 534 056	3 675 910	3 287 394	2 351 655	941 385	888 972	2 535 302
EIF2SL	P1	3 037 536	2 848 414	2 242 734	2 247 032	3 103 092	3 172 973	3 388 775	3 367 174
	P2	1 797 420	1 671 103	1 420 188	1 688 400	705 314	1 191 184	1 319 538	1 837 027
	P3	1 748 696	1 432 263	1 542 417	1 560 834	1 421 643	2 063 832	810 511	1 766 026

Supplementary Figure S10: Western blots and intensity of bands related to Figure S7

Supplementary Table S1: IC50 of the different cell lines used in this paper

IC50 based on MTT results (96 hours) supported by data from PI staining (48 hours). Correlated to Figure 2A.

Cell line	IC50 (μM)	Source
TK13	8	Current study
TK14	8	Current study
TK16	6	Current study
TK9	10	Current study
TK10	>10	Current study
TK12	>10	Current study
TK18	>10	Current study
AMO-1	8	Current study
AM0-CFZ	4	Current study
JJN3	6	Müller et al, 2013 [13]
RPMI8226	7	Müller et al, 2013 [13]

Supplementary Table S2: Proteins pulled down from ATX-101 sensitive TK cell lines

Protein pulled down using the MIB assay from three biological replicas of untreated TK13, 14 and 16 (39 proteins). Correlated to Figure 7.

Mucin-1	MUC1
THO complex subunit 1	THOC1
Calcineurin-binding protein cabin-1	CABIN1
Conserved oligomeric Golgi complex subunit 4	COG4
P-selectin glycoprotein ligand 1	SELPLG
Leukocyte immunoglobulin-like receptor subfamily B member 4	LILRB4
Protein LSM14 homolog A	LSM14A
Lymphoid-restricted membrane protein	LRMP
Isoform 11 of Transcription factor 4	TCF4
PR domain zinc finger protein 1	PRDM1
Protein disulphide-isomerase	PDIA4
Pleiotropic regulator 1	PLRG1
Rho GTPase-activating protein 4	RHG04
Chromosome 11 open reading frame 58	C11orf58
Tumor susceptibility gene 101	TSG101
BRISC and BRCA1-A complex member 1	BABAM1
Protein FAM32A	FAM32A
Zinc finger protein 428	ZNF428
Na(+)/H(+) Exchange regulatory cofactor	NHE-RF1
Isoform 3 of Acyl-CoA 6-desaturase	FADS2
Integrin beta-1	ITGB1
Isoform 2 of Tyrosine-protein kinase Lyn	LYN
Lysosomal alpha-glucosidase	GAA
Coronin-1A	CORO1A
Isoform 2 of Tumor protein D52	TPD52
High mobility group nucleosome-binding domain-containing protein 5	HMGN5
Isoform 2 of Tumor necrosis factor receptor superfamily member 17	TNFRSF17
Isoform 2 of Peroxisomal bifunctional enzyme	EHHADH
Transcription initiation factor TFIID subunit 10	TAF10
E3 ubiquitin-protein ligase ZNRF2	ZNRF2
Myeloid-derived growth factor	MYDGF PE
Cell growth regulator with EF hand domain protein 1	CGREF1PE
Vesicle-associated membrane protein 8	VAMP8
Isoform 2 of Regulator of nonsense transcripts 3B	UPF3B
Isoform 2 of Serine/threonine-protein kinase D2	PRKD2
Differentially expressed in FDCP6 homolog	DEF6
U3 small nucleolar ribonucleoprotein protein IMP3	IMP3
Nuclear receptor-binding protein	NRBP1
